# Immunological synapse: structures, molecular mechanisms and therapeutic implications in disease

**DOI:** 10.1038/s41392-025-02332-6

**Published:** 2025-08-11

**Authors:** Zheng Chao, Qi Mei, Chunguang Yang, Jing Luo, Peikun Liu, Hao Peng, Xiangdong Guo, Zhinan Yin, Le Li, Zhihua Wang

**Affiliations:** 1 Department of Urology, Tongji Hospital, Tongji Medical College, Huazhong University of Science and Technology, Wuhan, China; 2 Institute of Organ Transplantation, Tongji Hospital, Tongji Medical College, Huazhong University of Science and Technology, Key Laboratory of Organ Transplantation, Ministry of Education, NHC Key Laboratory of Organ Transplantation, Key Laboratory of Organ Transplantation, Chinese Academy of Medical Sciences, Wuhan, China; 3 Cancer Center, Shanxi Bethune Hospital, Shanxi Academy of Medical Science, Tongji Shanxi Hospital, Third Hospital of Shanxi Medical University, Taiyuan, China; 4 Cancer Center, Tongji Hospital of Tongji Medical College, Huazhong University of Science and Technology, Wuhan, China; 5 Institute of Reproductive Health, Center for Reproductive Medicine, Tongji Medical College, Huazhong University of Science and Technology, Wuhan, China; 6 The Affiliated Guangdong Second Provincial General Hospital of Jinan University, Guangzhou, Guangdong China; 7 State Key Laboratory of Bioactive Molecules and Druggability Assessment, The Biomedical Translational Research Institute, Health Science Center (School of Medicine), Jinan University, Guangzhou, China; 8 Taikang Tongji (Wuhan) Hospital, Wuhan, China

**Keywords:** Tumour immunology, Tumour immunology

## Abstract

The immunological synapse (IS) serves as the fundamental architectural framework for direct interactions and secretory crosstalk between immune cells, as well as between immune cells and other cells. Its dysregulation is thought to be a key underlying cause of immune evasion or inflammation observed in various diseases, including tumors and infections. Numerous recent studies have addressed key signaling mechanisms and reported novel targets related to IS, further broadening our understanding of its function and regulatory factors. However, a comprehensive review that highlights recent progress and consolidates past knowledge is still lacking. In this study, we delineated the pre- and postsynaptic structures constituting the IS between T cells, natural killer (NK) cells, dendritic cells (DCs), and macrophages. We also detail the specific signaling mechanisms and pathways that modulate the formation and disassembly of the IS, including cytoskeletal remodeling, membrane reshaping, integrin signaling, and force transduction. Following these experimental findings, we systematically review the central roles of IS in maintaining homeostasis and health and outline various diseases arising from IS disorders. Finally, we thoroughly explore targets and treatments related to IS on the basis of preclinical evidence and clinical trials, with the aim of providing further investigatory and therapeutic insights for researchers and clinicians.

## Introduction

Immune cells within the body are constantly exchanging information to regulate the immune system and maintain the health of the organism. At the heart of these dynamic interactions lies the IS, a structural foundation analogous to its neuronal counterpart.^1^ Research on the IS has historically focused on its composition and regulatory mechanisms due to the complexity of its formation, the numerous signaling pathways involved, and the intricate molecular events at the synaptic interface.^2–7^ This has only recently led to a preliminary comprehensive understanding of the IS. Currently, it is considered an essential checkpoint for various immune responses, widely controlling immune surveillance and host defense against tumors and pathogenic substances,^8–10^ coordinating the magnitude and scope of immune reactions,^11,12^ and even mediating immune tolerance^13–15^ while assisting in neural development.^16^

Unexpectedly, researchers have observed IS dysfunction in many diseases, prompting investigations into the specific connections between IS dysfunction and various conditions, including tumor occurrence,^17–19^ tumor metastasis,^20^ treatment resistance,^21^ infectious diseases,^22^ autoimmune diseases,^23^ noninfectious inflammation,^24^ and neurodegenerative diseases.^25^ Research has shown that immune dysfunction, leading to either immune deficiency or overactivation, may be the “culprit” behind the development of certain diseases rather than merely being a “participant”. Inspired by these findings, some researchers have directly intervened in key molecular targets that cause IS dysfunction or targeted the structural assembly of the IS, achieving unexpected therapeutic effects.^26–30^ For example, owing to the structural modification of the IS, DuoBody-CD40 × 4-1BB bispecific T-cell engagers (BiTEs) significantly enhance the DC/T-cell IS, thereby improving the anticancer capabilities of tumor-infiltrating lymphocytes in patients.^31^ These studies have reignited interest in exploring new treatment methods centered on the IS, which has further promoted new understanding of the signaling pathways, regulatory mechanisms, and intervention targets associated with the IS.

To provide researchers with a comprehensive understanding of the functions and characteristics of the IS and to keep them abreast of the latest developments, this review offers a thorough summary of recent research on the IS and systematically reviews its historical context. In this review, we describe the structures and components of the IS in the context of pre-synaptic and post-synaptic cells. Subsequently, we discuss the signaling pathways involved in the formation and disassembly of the IS. Specifically, we summarize four widely accepted regulatory mechanisms, namely, cytoskeletal remodeling, membrane reshaping, integrin signaling, and force signaling, which regulate the IS under physiological or pathological conditions. We then outline the functional roles of the IS in various physiological activities and maintaining health and elucidate the various diseases mediated by its dysregulation. Finally, this review summarizes the various preclinical drugs, treatment methods, and clinical trials targeting the IS and their effects on various diseases.

## History and milestone events in research on immunological synapses

The IS has a rich history, originating in the 1990s when immunologists noted that the interface of interaction between T cells and B cells resembled a “neuronal synapse.” This observation led to the adoption of the term “immunological synapse” within a niche community.^32,33^ It was soon formally defined as a molecular machine that regulates T-cell activation.^34^ Additionally, NK cells were found to possess an IS,^35^ and in 2000, the role of this structure in NK cell-mediated immune surveillance was first described.^35,36^ In the same year, immunologists proposed the idea of engineering artificial antigen-presenting cells (APCs) on the basis of the structure of the IS, envisioning future applications in tumor immunology, viral infection immunology, and immune tolerance.^37^

Entering the twenty-first century, the immunological community focused on the specific mechanisms regulating the formation of the IS. After nearly a decade of exploration, researchers discovered that the key regulatory mechanisms of IS formation primarily include the polarization of cellular membrane lipid rafts,^38–40^ the organization of membrane proteins,^2–4^ the remodeling of the cytoskeleton,^5–7^ and the activation of protein kinase signaling and DOCK.^41,42^ In 2003, it was first discovered that lytic synapses and stimulatory synapses have different activation thresholds.^43^ In addition to basic research into these mechanisms, scholars have sought to understand the role of the IS in maintaining homeostasis. Neuroscientists reported that microglia, important immune cells in neuro-immune interactions, can clear waste from the nervous system by phagocytosing neuronal synapses,^44^ leading to the introduction of the concept of the neuro-immunological synapse in 2003.^45^ Shortly thereafter, it was discovered that the immature IS of maternal decidual NK cells in early pregnancy protects fetal trophoblast cells from immune attack, indicating that the human body utilizes different stages of the IS for immune tolerance.^13^

Moreover, the associations between IS dysfunction and clinical diseases have gradually been revealed. In 2000, it was discovered that IS dysregulation occurs in Wiskott–Aldrich syndrome, primarily due to mutations in the Wiskott–Aldrich syndrome protein (WASP).^46^ This finding was the first explicit proposal of the critical regulatory role that cytoskeletal-associated proteins play in IS formation, sparking further discussions regarding other cytoskeletal regulatory mechanisms.^47^ Shortly thereafter, researchers reported that blocking ICAM-1 signaling in diabetic mice could inhibit T-cell expansion by preventing the formation of ISs, thereby delaying the progression of diabetes.^48^ Between 2003 and 2006, several reports highlighted the role of the IS in various conditions, including graft-versus-host disease (GVHD),^49^ autoimmune diseases,^50^ virus infections^51^ (such as HIV^22,52^ and Kaposi’s sarcoma-associated herpesvirus^53^), inflammatory bowel disease,^54^ and metabolic disorders.^55^ In vitro experiments and mouse models have also demonstrated the importance of the IS in anti-tumor responses^56^ and revealed that lymphoma cells could exchange membrane components through homotypic synapses, facilitating the spread of leukemia.^57^ In 2005, it was first proposed that IS function could be enhanced using BiTEs to overcome tumor immune evasion.^58^

Entering the 2010s, advancements in imaging technology allowed researchers to directly observe the structure of the IS in vitro in cultured immune cells using super-resolution microscopy.^59^ Additionally, techniques for in vivo imaging of virological synapses were developed.^60^ Since 2020, the rapid development of spatial omics and flow cytometry imaging technologies has further facilitated direct investigations of the associations between the IS and diseases in human disease samples.^61,62^ Another trend that began to shape IS research around the 2010s was its relationship with mechanics. In 2009, it was proposed that the T cell receptor (TCR) itself functions as an anisotropic mechanosensor.^63^ Research has revealed that mechanical signals are essential for TCR activation and IS formation.^64,65^ In 2016, it was discovered that synaptic forces promote the destruction of target cells,^66^ highlighting that the killing of target cells by cytotoxic T lymphocytes (CTLs) can be enhanced by increasing the force applied at the synaptic interface. This finding shifted the focus of subsequent research toward identifying new mechanical mechanisms underlying immune evasion in tumor cells and developing therapies targeting the IS on the basis of its mechanical properties.

**Fig. 1:**
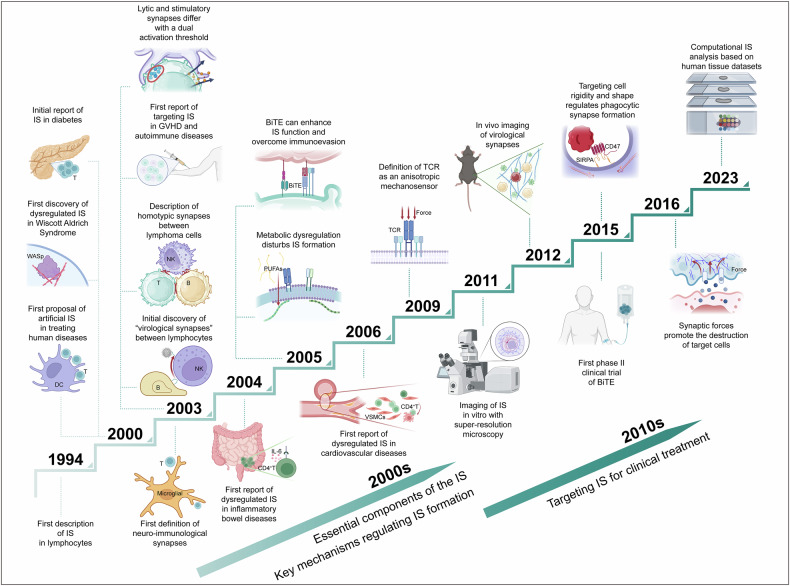
History of research on immunological synapses. This figure illustrates the major discoveries related to immunological synapses (IS), from their initial formal reporting and roles in diseases to the identification of key regulatory mechanisms and treatment inspirations. It highlights the progress made over time in understanding the structure and regulatory mechanisms of the IS during the 2000s. With the advent of new technologies, the direct observation of IS images both in vivo and in vitro in the 2010s has significantly enhanced our understanding of IS and has influenced clinical treatment approaches. Image created with BioRender (https://biorender.com/)

The first phase II clinical trial of BiTEs started in 2015,^67^ and in 2017, CD200R immune regulatory fusion proteins were reported, in which the cytoplasmic tail was replaced with the signaling domain of the costimulatory receptor CD28.^68^ Importantly, the progress of IS research over the past few decades has been rapid (Fig. 1). In the current landscape, where various high-tech techniques are continually emerging and the transition from basic to clinical research is increasingly valued, the future of IS research is expected to focus on two main directions: (1) utilizing spatiotemporal omics methods to analyze the mechanisms of action and key intervention targets of the IS in various diseases and (2) developing new immunotherapies based on the enhanced understanding of the IS structure.

## Structures and components of immunological synapses

Uniting cellular contact with the intricate transmission of signals, the IS initially delineated T cell-APC connections, but they have since been expanded to encompass a variety of immune cell interactions, including NK cell lytic and inhibitory synapses,^35^ T cell stimulatory and lytic synapses,^69,70^ NK cell‒DC synapses^71^ and phagocytic synapses of macrophages.^72^ However, the fundamental criteria for the IS remain relevant and include direct immune cell–target cell contact, immune cell polarization, and the orchestration of productive signaling at the synaptic interface.^73^

Notably, IS formation is characterized by a triphasic nature: initiation, effector, and termination. The initiation phase involves interactive encounters between effector cells and potential target cells, catalyzed by binding and adhesion receptors, ultimately culminating in the engagement of activation receptors, such as the TCR, with cognate antigen-presenting molecules encoded by major histocompatibility complex (MHC) genes.^74^ During the effector stage of lytic synapses, cytotoxic T cells undergo a series of molecular and cellular rearrangements that enable them to effectively eliminate their target counterparts.^75^ These processes involve critical and highly coordinated mechanical actions, such as signal transduction via dynamic microclusters formed by activated receptors, filamentous actin (F-actin) polymerization at the IS, granule convergence, and polarization of the microtubule organizing center (MTOC)^76,77^ Thus, precise temporal coordination is essential for achieving optimal cytolytic capabilities.

The culmination of the IS is observed in the termination stage and is characterized by the detachment of effector cells from deceased target cells.^78^ This step is crucial for maintaining immunological homeostasis, as failure to detach could lead to prolonged immune responses and inflammation.^78^ Notably, cytotoxic cells, both in vivo and in vitro, exhibit rapid and efficient sequential killing capacities; however, T cells and NK cells display considerable heterogeneity in this regard.^79,80^ It is imperative to delineate the distinct immune cells on either side of the synapse, as this can help readers gain a comprehensive understanding of the structure and composition of the IS. The same immune cell, such as a T cell, may function as a pre-synaptic cell capable of lysing target cells, as well as a post-synaptic cell receiving antigen presentation from APCs. We will discuss the roles of these immune cells separately.

### T cells

#### Post-synaptic T cells

T cells play a pivotal role in adaptive tumor immunity. Initially, primed T cells, which are typically activated by APCs, orchestrate the proliferation and differentiation of T cells, amplifying and modulating the immune response. These activated T cells subsequently interact with tumor cells or other components of the immune system to execute their effector functions.^81^ During their migration through peripheral lymphoid organs, T cells exhibit remarkable motion and employ specific mechanisms to engage with APCs, surveying the presence of particular ligands in the surrounding environment.^82^ Upon encountering APCs that present compatible peptide-MHC (pMHC) complexes, T cells cease their movement and orient themselves toward the APCs to form the IS.^83^ This process occurs within a specialized domain known as the “stimulatory synapse,” where localized curvature of the T cell membrane at the nanoscale facilitates sustained interactions involving relatively small TCR‒pMHC complexes, amidst the extensive surface receptors and adhesion molecules of T cells and APCs.^84^

Moreover, T cell activation requires co-stimulatory signals, interactions with adhesion molecules, and the initiation of mechanical signals within this confined space. Modulating the stiffness of the stimulatory synapse via the use of responsive hydrogels and DCs expressing altered cytoskeletal proteins has the potential to reduce the stimulus dosage required for T cell activation.^85^ Notably, compared with CD8^+^ T cells, CD4^+^ T cells are more reliant on stiffness.^85^ Furthermore, when T cells are stimulated with pMHC instead of anti-CD3ε, the response to stiffness is heightened, suggesting a mechanosensory mechanism involving receptor deformation.^85^ Recent studies have also proposed that membrane curvature and the exclusion of CD45 are pivotal for triggering TCR signals and facilitating T cell activation.^86,87^ Research has indicated that the butyrophilin protein BTN3A1 inhibits the activation of tumor-reactive αβT cell receptors by hindering the N-glycosylation of CD45, leading to the retention of CD45 at the IS.^88^ Notably, CD277-specific antibodies synergistically restore αβT cell effector functions and enhance BTN2A1-mediated γδ lymphocyte cytotoxicity against BTN3A1^+^ cancer cells, thereby impeding malignant progression.^88^

**Fig. 2:**
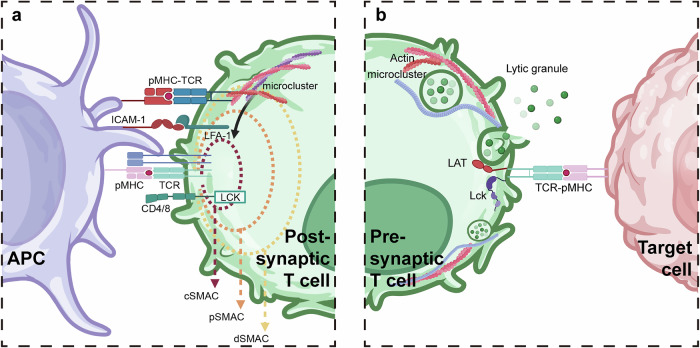
The IS interface between T cells and APCs. **a** When T cells bind to APCs, they form a stimulatory synapse characterized by three concentric bullseye-like structures on the T cell surface. The cSMAC contains TCRs bound to pMHC complexes and signaling molecules. The pSMAC and dSMAC contain adhesion molecule pairs. When pMHC forms a complex with TCR in the dSMAC, it can induce the formation of microclusters, which will pull the distal pMHC-TCR complexes to move to the cSMAC. **b** When TCR on CTLs are activated by pMHC complexes, they establish a lytic synapse. Through a contractile actin meshwork rich in myosin, CTLs exocytose lytic granules to release granzymes and perforin toward the target cell. Image created with BioRender (https://biorender.com/)

The molecular organization within T cells is intricately structured in a three-layer concentric “bull-eye” configuration, with the central supramolecular activation cluster (cSMAC) surrounded by two concentric rings primarily composed of an antigen-bound TCR and associated signal transduction molecules. Peripheral SMAC (pSMAC) and outer distal SMAC (dSMAC) are enriched in adhesion molecule pairs.^89^ The dSMAC-based pMHC-triggered TCR can induce F-actin-dependent accumulation of receptors into small clusters, known as microclusters, which then move centripetally toward the cSMAC.^90,91^ Interestingly, recent studies have reported the existence of a transient cell-extracellular matrix (ECM) compartment in the synapse region, known as exo-cSMAC. The exo-cSMAC is composed of vesicles containing TCRs that are internalized by the same APCs, thereby promoting communication and exchange between APCs and T cells (Fig. 2).^92^ Once cellular contacts are established, antigen receptors and adhesion molecule pairs synergistically constitute the adhesion and stimulatory interface. TCRs promote various aspects of integrin function through inward signaling, whereas reciprocally, ligand-bound integrin signaling propagates outward co-stimulatory signals, thereby lowering the threshold for antigen-driven lymphocyte activation.^93^

#### Pre-synaptic T cells

Postactivation, CD8^+^ T cells differentiate into CTLs, which are critical components of the immune response against tumor cells. These CTLs contain cytotoxic substances within lytic granules (LGs) and undergo exocytosis during TCR signaling, releasing granzymes and perforin in a rapid and highly localized manner at a region known as the immune or lytic synapse.^94^ These events typically occur in the annular region between the center and periphery of the IS, which is characterized by a contractile actin meshwork rich in myosin that forms part of the pSMAC. Furthermore, CTLs possess a remarkable capacity for serial cytotoxicity, detaching from a target cell after elimination and efficiently moving on to the next target.^95,96^ This capability is supported by the fact that CTLs can be activated by as few as two to three specific pMHC complexes present at the IS interface, enabling them to release cytotoxic granules effectively and deliver a lethal strike within a brief timeframe.^97,98^ During immune responses, CTLs utilize these mechanisms to efficiently eliminate targets while navigating through pathological tissues.^99,100^ The combination of multiple killing agents enhances the cytotoxic response, ensuring that CTLs can effectively surveil potentially hazardous cells. However, only partially related mechanisms have been elucidated thus far.

LG exocytosis requires a series of steps: tethering, docking/priming, and ultimately fusion with the target cell membrane. After exocytosis, the granule membrane undergoes recycling in preparation for the next killing event.^81,101^ Functional analyses indicate that, following synaptic cleft formation, CTLs initially release soluble single-core granules (SCGs) containing perforin to fill the synaptic gap, followed by the release of multi-core granules (MCGs) in the form of supramolecular attack particles to delay the killing of resistant target cells.^102^ The activity relies on WASP and the Arp2/3 actin nucleation complex, which are essential for generating synaptic forces and facilitating efficient cytotoxicity while also mediating the physical deformation of the target cell surface during CTL‒target cell interactions, leading to membrane damage.^103^

Lattice light-sheet microscopy of CTL-target cell conjugates revealed that, at mature synapses, F-actin is cleared from the central supramolecular activation cluster (cSMAC) prior to the release of LG. The release of LG subsequently triggers the restoration of F-actin at the lytic synapse, thereby preventing further LG exocytosis and serial killing;^100,104^ however, more in-depth analyses using super-resolution microscopy have shown that cSMAC is not devoid of F-actin but rather contains a low-density actin network.^105^ Upon cell activation, the size of the pores formed is compatible with LG docking and fusion at the plasma membrane.^77^ Thus, actin plays a dual role in the secretion process, depending on the maturity stage of the lytic synapse: in immature synapses, a dense actin cortex inhibits secretion, whereas in mature synapses with a low-density actin network, nanoscale actin filament dynamics fine-tune LG exocytosis. Following CTL attack, the formation of perforin pores in the target cell membrane facilitates the entry of granzyme B and other serine proteases, ultimately inducing rapid apoptosis of the target cell.

Notably, despite the close spatial proximity of stimulating synapses and lytic synapses with target cells, their activation thresholds differ significantly.^10^ Lytic synapses can occur at both low and high antigen concentrations, whereas stimulating synapses only form when the target cell provides strong antigen stimulation.^43^ This spatial and temporal dissociation between IS and LG secretion is rational and essential. On the one hand, the polarization of LG and TCR signaling activation occurs before the mature stimulating synapse is formed, endowing CTLs with robust cytotoxic functionality prior to the extensive accumulation of TCR/CD3.^4^ On the other hand, CTLs can eliminate multiple targets encountered simultaneously by polarizing LGs toward various opposing targets without discriminating their antigenic potential, ensuring a highly sensitive, rapid, and effective cytotoxic response triggered by the recognition of minute amounts of specific pMHC complexes on the surface of target cells.^106^ The intricate process of multi-polarization not only enables CTLs to combat viral infections but also empowers them to effectively counter tumor growth by eliminating innocent targets.^10,107^ In contrast, CD4^+^ helper T cells interact concurrently with diverse APCs and polarize their Golgi apparatus toward the APC, providing the strongest stimulation, unlike CD8^+^ T cells, which polarize toward multiple targets without discrimination.^108,109^ These contrasting behaviors collectively contribute to an optimal adaptive immune response: helper T cells provide selective assistance, whereas CTLs swiftly neutralize heterogeneous targets.^10,110^ Following multiple killing events, CTLs can transition from rapid granzyme B-mediated killing to slower death receptor-mediated killing, thereby extending their overall killing capacity.^111^

In addition to classic receptor‒ligand interactions, recent studies have underscored the role of newly discovered receptor‒ligand interactions and structures within the T cell IS.^112,113^ NK cell granule protein 7 (NKG7), which is highly expressed on NK cells, has been found to enhance the degranulation of T cells.^112^ However, T cells lacking NKG7 form prolonged ISs with tumor cells, leading to increased secretion of inflammatory cytokines and compensating for the reduced cytotoxic effects.^112^ These findings suggest that NKG7 may serve as a switch target to accelerate direct cytotoxicity while limiting potentially harmful inflammatory reactions. Another study highlighted the importance of the cis-structure of the CD28-B7 ligand pair for T cell activation signals; disruption of this structure, which is shaped by phosphatidylinositol 3-kinase (PI3K)-driven membrane reconstruction, significantly hampers the cytotoxicity of CD8^+^ T cells both in vitro and in vivo.^113^

### NK cells

NK cells are prototypical innate lymphocytes and serve as the first line of defense against tumor cell transformation, growth, and metastasis.^114^ They exert their functions primarily by releasing cytolytic components, such as perforin and granzymes, or by expressing ligands that activate death receptors on target cells. Additionally, NK cells can secrete various cytokines and chemokines to recruit and activate other immune cell types.^115^ Like other immune cells, most NK cell effector functions require direct cell‒cell contact with their target cells.^116^

Compared with CTLs, NK cells exhibit shorter and more dynamic interactions with tumor cells.^117^ Despite these findings, both cell types demonstrate similar effectiveness in killing target cells, suggesting that these kinetic differences may stem from distinct activation mechanisms and signaling pathways, such as calcium influx, rather than differences in their ability to induce cell death.^117^ NK cell cytolytic activity is triggered by the recognition of surface molecules that exhibit stress or transformation features, including MHC-like molecules and UL16-binding proteins (ULBPs), which engage the TCR and the activating NK receptor NKG2D, respectively.^118^ The formation of the NK cell IS mirrors that of T cells, involving multiple sequential steps: initial transient contact for target cell surveillance, firm adhesion mediated by adhesion molecules and cytoskeletal remodeling, and polarization and exocytosis of LG to induce target cell lysis.^119,120^ Thus, remodeling of the cell membrane topology and cytoskeletal structures is a prerequisite for effective NK cytotoxicity. For example, super-resolution microscopy has revealed that lenalidomide can increase the periodicity of actin filaments in the NK cell IS, leading to nanoscale rearrangements of the actin network, which enhances NK cell cytotoxic function.^121^ In contrast, alterations in the NK cell membrane structure may contribute to tumor cell evasion of immune surveillance. In tumor patients, intratumoral NK cells exhibit fewer membrane protrusions than liver and peripheral NK cells do, impairing their ability to recognize tumor cells, form lytic synapses, and execute cytotoxic functions.^122^ Further investigations revealed that these membrane changes are linked to altered sphingomyelin content in intratumoral NK cells as a result of disrupted serine metabolism within tumors.^122^ These findings suggest that targeting the shared structural basis of NK and T cells could provide a dual-benefit therapeutic strategy.

Recent research has shown that NK cell cytoskeletal remodeling and other IS-related events are governed by the complex integration of receptor signals.^123^ Unlike T cells, NK cell activity is regulated by the balance between various germline-encoded inhibitory and activating cell surface receptors. As a result, NK cell synapses are classified into two types, namely, activating NK immunological synapses (aNKISs) and inhibitory NK immunological synapses (iNKISs), which lead to distinct functional outcomes.

#### Activating NK immunological synapses

NK cell interactions with MHC I-negative target cells (e.g., tumor or virus-infected cells) lead to the formation of aNKIS. This process resembles the IS formed between T cells and tumor cells and involves several sequential steps: contact and adhesion, receptor engagement and signaling, actin cytoskeleton remodeling, receptor clustering and signal amplification, MTOC polarization, granule polarization and degranulation, and synapse disassembly.^124,125^ In this process, actin polymerization and accumulation at the aNKIS are essential for NK cell cytotoxicity, and disruption of the actin cytoskeleton severely impairs NK cell function.^126^ NK cell-activating receptors can be classified into two categories: (1) receptors that interact with peptides containing immunoreceptor tyrosine-based activation motifs (ITAMs), such as CD3δ, DAP12, and FcRγ, which include CD16, NKG2C/CD94, and natural cytotoxicity receptors (NCRs), such as NKp30, NKp44, and NKp46; and (2) receptors that interact with non-ITAM proteins, such as NKG2D, 2B4, or CD2.^127^ Most NK cells, along with some innate lymphoid cells, express the activating receptor NKp46, which is encoded by the NCR1 gene and plays a central role in the NK cell-mediated elimination of target cells.^128^ NKp46 recognizes ecto-calreticulin (ecto-CRT), which translocates from the endoplasmic reticulum (ER) to the cell membrane during ER stress.^12^ ER stress and ectopic CRT expression are markers of immunogenic cell death induced by chemotherapy or cellular senescence.^12^

Engagement of NKp46 with ecto-CRT triggers NK cell signaling, resulting in the formation of NKp46 microclusters in the IS.^12,129^ Low expression of NKp46 is correlated with reduced F-actin accumulation at the synapse, whereas overexpression of NKp46 in primary human NK cells enhances F-actin recruitment to the synapse, which is linearly correlated with granule polarization.^129^ Additionally, NKp46-mediated killing can be further enhanced by the ectopic expression of glycosylphosphatidylinositol (GPI)-anchored CRT.^12^

Importantly, NK cell activation does not necessarily involve the sustained participation of activating receptors. Observations of degranulation in individual NK cells have shown that repeated activation through the Fc receptor CD16 reduces perforin secretion, whereas perforin release can be restored upon subsequent activation through different receptors, such as NKG2D.^130^ However, repeated stimulation through NKG2D also diminishes perforin secretion, which cannot be rescued by CD16 stimulation.^130^ This phenomenon is attributed to CD16 shedding, which leads to synapse disassembly, promoting NK cell survival and continued killing of target cells. Pharmacological inhibition or expression of non-cleavable CD16 impairs NK cell motility and prevents separation from target cells, leading to a state of NK cell “exhaustion”, similar to what is observed in T cells.^130,131^

#### Inhibitory NK immunological synapses

In contrast to activating signals, inhibitory signals typically operate in the early stages of the IS. NK cells interacting with MHC I-positive (i.e., normal, healthy) target cells form iNKIS, which interferes with activating signals and stabilizes the synapse to prevent NK cell activation and target cell death.^11^ The formation of the iNKIS is distinct from that of activated NK immunological synapses (aNKIS) in that it lacks significant cytoskeletal remodeling and functions as an immune checkpoint. Furthermore, iNKIS formation is independent of ATP, lipid rafts, and the actin cytoskeleton and is instead driven by thermodynamic processes.^40,83,132,133^

iNKISs are composed primarily of inhibitory receptors containing cytoplasmic immunoreceptor tyrosine-based inhibitory motif (ITIM) domains, which can be grouped into two categories: (1) the immunoglobulin superfamily (e.g., KIR, LIR, and Siglec) and (2) C-type lectin family proteins (e.g., NKG2A/CD94). Interestingly, the number of inhibitory receptors in iNKIS is proportional to their overall expression levels and does not increase over time.^134^ Initially, inhibitory receptors cluster in the central region of the iNKIS, whereas adhesion molecules localize to the periphery.^134^ Over time, inhibitory receptors disperse throughout the iNKIS or form separate clusters, whereas lymphocyte function-associated antigen-1 (LFA-1) moves to the center and segregates from NK cell receptors.^135^ iNKIS is highly dynamic, with HLA-C/KIR patterns changing and interchanging over time, depending on the density of MHC-I and inhibitory receptors.^132,136^

The suppression of NK cytotoxicity necessitates a certain level of MHC-I expression; however, within the organism, the quantity of inhibitory receptors and the density of inhibitory receptor ligands on the cell surface are sufficiently high to engage all inhibitory receptors maximally.^137^ Consequently, when activating and inhibitory receptors are concurrently involved, the inhibitory signals predominate and override the activation of NK cells.^138^ KIR2DL5, a member of the KIR family, was recently identified as a binding partner for CD155.^139^ Immune infiltration of KIR2DL5^+^ cells is prominent in various types of CD155^+^ human cancers. The ITIM and immunoreceptor tyrosine-based switch motif (ITSM) domains of KIR2DL5 undergo tyrosine phosphorylation upon engagement, leading to downstream suppression of Vav1/ERK1/2/p90RSK/NF-κB signaling and inhibition of NK cell activation and tumor cell killing.^140^ Notably, KIR2DL5 binds to CD155 without competing with other known CD155 receptors, suggesting minimal technical challenges in developing monoclonal antibodies targeting this pathway.^140^ Another KIR family member, KIR3DL3, functions as an inhibitory receptor in CD56dim NK cells and terminally differentiated effector memory CD8^+^ T (CD8^+^ TEMRA) cells.^141^ Its ligand, HHLA2, is overexpressed in human cancers and is associated with poor prognosis.^142^ HHLA2 binding recruits KIR3DL3 to the IS, where it dampens activation signaling pathways such as NF-κB, thereby inhibiting CD8^+^ T cell and NK cell function and cytotoxicity. Given the widespread expression of HHLA2 in solid tumors, the KIR3DL3-HHLA2 pathway may be a key mechanism of tumor immune evasion.^141^

Additionally, Nogo receptor 1 (NgR1) has been identified as a novel inhibitory receptor in NK cells. While NgR1 has been reported to regulate actin cytoskeleton dynamics in neuronal synapses by activating the RhoA pathway, its role in tumor immunity has only recently been explored.^143^ NgR1 inhibits IS formation by modulating actin dynamics via the LIMK-cofilin pathway, which suppresses F-actin accumulation and granule polarization, thus reducing NK cell cytotoxicity. Conversely, NgR1 deficiency or blockade enhances NK cell contact stability with target cells and improves tumor control.^11^ This discovery expands our understanding of NK cell inhibitory receptors, suggesting that these receptors can regulate IS formation and cytoskeletal remodeling rather than solely interfering with activating receptor signals.^11^

### Macrophages

Macrophages are well known for their ability to phagocytose apoptotic cells, pathogens, and tumor cells. The interaction between macrophages and their phagocytic targets is akin to the contact between T cells, NK cells, and their targets, leading to the formation of an IS.^72^ However, there are significant differences between the global and local protrusions of T cell synapses and the phagocytic synapses of macrophages.^62,72^ During phagocytosis, membrane remodeling, cytoskeletal organization, and biochemical signaling are coordinated through mechanical signals driven by membrane tension.^144^ To extend pseudopodia over the surface of target particles during the early phase of phagocytosis, extensive membrane and cytoskeletal remodeling are needed. In the first phase, the pseudopods rapidly extend, with actin polymerization pushing the plasma membrane forward.^144^ In the second phase, once the membrane reservoir is depleted, membrane tension increases, directly influencing the organization of the Rho GTPase Rac1, phosphatidylinositol 3-phosphate (PI3P), and the cytoskeleton. Moreover, increased membrane tension triggers the exocytosis of vesicles containing GPI-anchored proteins, expanding the membrane surface and enhancing the efficiency of phagocytosis, particularly for larger particles.^144^

Macrophages recognize and internalize various complex targets via a wide range of phagocytic receptors.^145^ In tumor-specific antibody immunotherapy, stable conjugates form between tumor cells and macrophages expressing Fcγ receptors (FcγRs).^146^ Among these conjugates, actin, FcγR, and tyrosine phosphoproteins accumulate in multifocal SMACs. FcγRs are a class of phagocytic receptors that initiate phagocytosis by selectively binding to the Fc portion of IgG molecules on their targets.^147^ Macrophages form actin-rich phagocytic cups around their targets, and the extension and closure of these cups are critical for efficient phagocytosis.^148^ In FcγR-mediated phagocytosis, podosome-like structures serve as primary sites for F-actin polymerization, and their actin-rich cores are surrounded by integrin receptors, potentially functioning as mechanosensors that detect and respond to mechanical cues from the target, thus facilitating more efficient phagocytosis.^149,150^

Signal regulatory protein alpha (SIRPα, also known as SHPS-1 or CD172A) is another widely expressed phagocytic receptor in macrophages. The co-localization of SIRPα with FcγR inhibits cell activation after FcγR engagement, while their separation permits phagocytic activation.^151,152^ CD47, the weak-binding ligand for SIRPα, is known as a “self-marker” protein. Upon binding to SIRPα, CD47 prevents the separation of FcγR from SIRPα, inhibiting the formation of concentric FcγR rings and leading to the accumulation of SIRPα at phagocytic synapses.^152^ This inhibits the assembly of non-muscle myosin IIA, thereby suppressing the phagocytosis of “self” cells, including cancer cells.^153^ However, when CD47 is absent or blocked by antibodies, SIRPα relocates at the contact point between cells, reducing tyrosine phosphorylation levels and enhancing phagocytic efficiency.^154^ Moreover, in low-pH environments (e.g., pH 6), the membrane stiffens, reducing the synergy of self-signaling during the phagocytosis of cancer cells.^155^ During the phagocytosis of apoptotic cells, integrins collaborate with other receptors to mediate this process. For example, TIM-4-mediated phagocytosis requires the involvement of β1 integrins and activates integrin-dependent signaling through Src family kinases and focal adhesion kinase (FAK).^156^ Additionally, the interaction between β5 integrins and stabilin-2 promotes the engulfment of apoptotic cells.^157^ Similarly, the co-expression of αvβ5 and Mer increases Rac-1 activation, cytoskeletal reorganization, and apoptotic cell clearance.^158^

Like molecular segregation in the IS, exclusion at phagocytic synapses relies on the axial molecular size of CD45.^159^ Experiments with chimeric constructs have shown that shorter CD2/CD45 molecules can enter phagocytic synapses, whereas longer CD43/CD45 molecules are excluded.^159^ Integrins and cytoskeletal regulators are crucial in establishing exclusion zones beyond the IgG layer at phagocytic synapses. However, tumor-associated macrophages (TAMs) exhibit an intermembrane distance between integrins and their receptors ( ~ 30 nm), which is significantly greater than the 14 nm distance observed in immune receptors responsible for signal transduction (e.g., TCR/MHC-II and CD80/CD28).^159^ These findings suggest that integrin-mediated macrophage–target interactions promote receptor engagement at low ligand densities or with larger particles, stabilizing adhesion and promoting efficient signal transduction postphagocytosis.

Recent studies have revealed that phagocytosis is influenced not only by biochemical signals on the target surface but also by the target’s biomechanical properties.^160^ For example, macrophages exhibit a preference for rigid targets during phagocytosis, and rigid targets overactivate myosin II at the phagocytic synapse, suppressing “self” signals.^153,161^ Macrophages are mechanosensitive cells that respond to ECM rigidity by modulating their actin cytoskeleton, which in turn affects cell morphology, membrane topology, and the migration of immune complexes.^162^

Macrophages can form IS not only with phagocytic targets but also with T cells, NK cells, and B cells.^162–164^ The lytic synapses between NK cells and lipopolysaccharide (LPS)-activated macrophages likely require circular F-actin accumulation to facilitate directed granule secretion.^163^ After a 10-minute co-culture with LPS-activated macrophages, ICAM-1 can be clearly observed within the lytic synapses of NK cells and macrophages.^163^ Additionally, macrophages can enhance NK cell cytotoxicity by promoting NK cell proliferation, cytokine secretion, and the expression of NK cell activation receptors.^163^

### Dendritic cells

DCs are professional APCs that play crucial roles in T-cell activation. They capture antigens, process them intracellularly, and present them to T lymphocytes by forming synapses^165,166^. Unlike other immune cells, the interaction between DCs and T cells involves not only synapse formation for signal capture but also a “licensing” process in which DCs receive reciprocal signals^9,167^. Upon CD40-CD40L interaction, postsynaptic DCs (psDCs) further enhance the formation of the IS by upregulating MHC-I expression, promoting their maturation and activation, and improving their migratory capabilities^168^.

DCs have a relatively short lifespan, making it crucial to maintain their viability after completing antigen presentation. Studies have shown that the formation of the IS provides DCs with anti-apoptotic signals.^166^ The recruitment and activation of prosurvival proteins, such as Akt, help initiate pathways that activate NF-κB while inhibiting proapoptotic factors ^such as FOXO281^. Multiphoton imaging studies revealed that, in the absence of antigens, interactions between T cells and DCs are brief (less than 3 min), whereas antigen recognition significantly extends the contact duration to 3 ~ 5 h, promoting T cell activation and proliferation.^169^ The IS formed between DCs and CD8^+^ T cells differs from the classic SMAC structure, particularly in in vitro cultures.^170,171^ Non-conventional DCs (nsDCs) can form multifocal synapse structures characterized by TCR clustering at multiple sites on the synaptic membrane, whereas the DC side exhibits a multifocal topology with actin patches separating these sites.^172^ Although this multifocal structure enhances antigen presentation efficiency, the mechanisms by which nsDCs coordinate TCR signaling remain unclear.^173^

During the formation of DC synapses, ICAM-1 aggregation promotes the clustering of LFA-1 on T cells, concentrating MHC-II on the DC surface in contact with T cells.^85,174,175^ DC maturation is accompanied by cytoskeletal remodeling, which increases cortical stiffness, opposing the action tension on LFA-1 and enhancing adhesion, facilitating T cell activation.^174^ While these processes have been partially elucidated, the triggers and mechanisms underlying actin remodeling in DC synapses are still not fully understood. Some researchers have proposed that the migration of immature DCs relies on two distinct pools of actin: a RhoA-mDia1-dependent pool at the rear for forward movement and a Cdc42-Arp2/3-dependent pool at the front to limit migration and enhance antigen capture.^176^ DCs switch between these different actin nucleation mechanisms during migration and antigen uptake. However, during the formation of the T-DC IS, the cytoskeleton is remodeled by the WAVE regulatory complex (WRC). Actin polymerization at the dSMAC supports myosin arc formation, colocalizing with LFA-1 and enabling TCR movement within the pSMAC to ensure proper T-cell stimulation and activation.^177^

WRC also regulates F-actin depolymerization and renewal at the DC synapse, with actin depolymerization driving the transformation of the T cell synapse from a multifocal to a unifocal structure.^170,171^ In the absence of WRC, the assembly of the DC cytoskeleton is compensated for by formin-mediated actin polymerization. However, the inability to depolymerize the actin network impedes synapse turnover, reducing T cell activation.^170,177^ This highlights that while a stable IS is crucial for initial T cell activation, overly stable synapses may hinder the ability of DCs to activate additional naïve T cells, thereby impacting overall immune response efficiency.^170,177^

In addition to interacting with T cells, DCs can form regulatory ISs with NK cells, which are essential for coordinating and modulating anti-tumor immune responses.^178,179^ DCs not only influence resting NK cell function but also help naïve NK cells acquire effector functions by increasing NK cell proliferation, cytokine secretion, and cytolytic activity.^179–182^ The interaction between mature DCs and NK cells is accompanied by increased calcium flux within NK cells, further promoting synapse formation.^183^ This process depends on actin cytoskeletal integrity, lipid raft accumulation, and microtubule stability.^180^ Disruption of these structures significantly impairs the phosphorylation of proteins at synapses, thus inhibiting NK cell activation by DCs.^180^ Reciprocally, DC-activated NK cells can further promote DC maturation, cytokine production, and survival.^183,184^ NK cells also selectively kill immature DCs to ensure that only fully mature DCs remain to initiate robust T cell responses, which is considered a key step in triggering effective anti-tumor immune responses.^71,185^

## Signaling and molecular mechanisms regulating immunological synapse formation and disassembly

The formation and disassembly of the IS are highly complex processes that encompass both the intracellular signaling of pre-synaptic and post-synaptic cells throughout the entire lengthy process, as well as the information exchange between the two cells, thus involving numerous signaling pathways. In summary, during the “preparation phase,” chemokines such as CCL2, CCL5, and CXCL12, along with extracellular vesicles (EVs) carrying TCRs and antigens, initiate “priming signals” in immune cells, endowing them with increased mobility to seek their targets.^167,186–188^ In the “contact phase,” T and NK cells first interact with their ligands through their activating/inhibitory receptors, triggering downstream activating/inhibitory signals such as the ZAP70 and LCK signals downstream of the TCR and the PKC, ITK, and PLCγ signals downstream of CD3/28, accompanied by the interaction of integrins and adhesion molecules.^74^ This leads to the activation of relevant stimulatory or inhibitory signals within the cell and initiates characteristic molecular events such as cytoskeletal remodeling and centrosome polarization.^189,190^ Changes in the cytoskeletal structure result in the remodeling of the membrane structure and the transduction of mechanical signals.^191,192^ Importantly, these signaling pathways and molecular mechanisms are not triggered in a fixed sequential order, and sometimes they can occur simultaneously. In the “dissociation phase,” a weakening of the aforementioned signals is typically observed, and there are also new signaling mechanisms that promote dissociation, such as the detachment of CD16.^130^ In this review, we focus on elucidating four important signaling mechanisms involved in the assembly and dissociation of the IS: cytoskeletal remodeling, membrane reshaping, integrin signaling, and force signaling.

### Cytoskeletal control of immunological synapse formation and disassembly

The interplay among immune cells hinges on the perpetual reconfiguration of the cytoskeleton, an event that not only delineates the interface between T cells and APCs and the engagement zone of NK cells with their targets but also orchestrates an asymmetric redistribution of molecules and organelles within lymphocytes, ultimately leading to the transient establishment of polarity.^193^ Cytoskeletal rearrangement is one of the initial phenomena triggered by TCR signaling, where temporal and spatial coordination of the cytoskeleton, cell surface receptors, and intracellular signaling proteins unfolds at the IS. This enables the integration of mechanical cues, biomechanical signal transmission, and the directed secretion of effector molecules.^194,195^ The formation of the synaptic F-actin ring results in the polarization of the centrosome, Golgi apparatus, endosomes, secretory vesicles, multivesicular bodies, and mitochondria toward the IS interface.^196–198^ This process involves signaling cascades, including the production of phosphatidylinositol trisphosphate by PI3K, which summons DOCK2, an activator of the Rho family GTPase Rac1.^199^ Activated Rac1, in turn, triggers WAVE2 activation on the plasma membrane, facilitating the stimulation of the Arp2/3 complex and subsequent actin polymerization.^91,200^ The architecture and fluidity of this radiant dendritic actin network bear a striking resemblance to the structural and kinetic attributes evident in the lamellipodia of migrating cells, both of which are capable of generating a centripetal flux of actin, commonly referred to as retrograde actin flow.^201,202^ Given its pivotal contribution to the formation of the IS, the polymerization of F-actin at the synapse serves as a hallmark of the stability and functional integrity of the lytic IS in both T and NK cells. Dysfunction in actin polymerization at the IS, often stemming from deficiencies in actin nucleation-promoting factors such as WASP, can precipitate profound immune deficiencies, distinguished by the presence of anomalous or compromised cytotoxic cells.^203^

In CTLs, the initial engagement with APCs is facilitated by elongated T cell protrusions, which initiate finger-like contacts with the target cell.^204^ Intriguingly, these actin-rich extensions rapidly flatten as actin is depleted within the synapse, thereby expanding the contact area between the T cell and the target membrane.^204^ Following the establishment of the synapse, the deployment of granules is contingent upon the disassembly of cortical actin, which is propelled by the subsequent reassembly of actin.^104^ Additionally, a distinct category of actin-based protrusions, microvilli, has been identified on the surface of T cells.^205^ While these microvillar projections are a feature of resting T cells, recent research has broadened findings to encompass activated cells, as evidenced by the aggregation of TCRs at the tips of microvilli.^187,205^ Initially, T cell microvilli were perceived as sensors for antigen recognition. However, the revelation of T cell microvilli particles (TMPs) deposited on the APC surface implies a more versatile role for these structures as “immunological synapse bodies,” potentially serving as a novel mode of intercellular communication through the transfer of an unprecedented class of membrane vesicles.^187^ Upon contact with the APC, the T cell cortex undergoes a pronounced increase in the concentration of actin and the assembly of actin networks.^206^ The activation of a multitude of actin regulatory proteins is meticulously coordinated by the TCR, the integrin LFA-1, and the co-stimulatory receptor CD28 through a network of signaling pathways at the IS, thereby enhancing actin polymerization and optimizing TCR signaling, integrin activation, and T cell spreading across the APC surface.^175^ The profound cytoskeletal changes promoted by the TCR-mediated engagement of homologous pMHC complexes require coordinated rearrangement of the actin and microtubule cytoskeletons at the IS.^70,207^ This actin remodeling process commences within seconds following TCR stimulation and precedes the translocation of the centrosome to the IS, a process considered a definitive feature of T cell polarity during IS assembly.^208^ A recent study employing the Sas4/p53 knockout model to abrogate centrioles in CTLs has assessed the contribution of the centrosome to the cytotoxic processes of CTLs, suggesting that the deletion of Sas4/p53 in CTLs led to a decrease in cytotoxicity, although not its wholesale abrogation, as the fundamental MTOC and a somewhat disordered microtubule cytoskeleton were maintained.^189^ However, the absence of Sas4/p53 results in the precipitation of many intracellular perturbations, including the depletion of granule content and aberrations in actin remodeling, both of which significantly compromise cytotoxicity.^209^ This, along with other studies, underscores the pivotal role of the centrosome‒microtubule network in orchestrating the polarized transport of granules to their designated secretory destinations with precision^196^. In a similar vein, DCs undergo repositioning of the centrosome at the DC‒T-cell interface following Toll-like receptor (TLR) engagement, which is instrumental in facilitating polarized secretion.^210^ Investigations into B lymphocytes have also revealed that the centrosome is encircled by focused reticular F-actin, which serves as a fundamental function of this F-actin reservoir by securing the centrosome to the nucleus via the nuclear skeleton and the linker of the nucleoskeleton and cytoskeleton (LINC) complex.^211^ The upstream polarization of the centrosome to the IS shifts the equilibrium between F-actin assembly and disassembly, leading to the localized depletion of centrosomal F-actin and promoting its detachment from the nucleus and subsequent migration to the IS^190^. In B cells, this occurs through reduced recruitment of the centrosomal actin nucleator Arp2/3, promoting its synaptic localization. Another study exploring the molecular patterns of antigen extraction in B cells revealed that repositioning of the centrosome, in conjunction with changes in microtubule dynamics, facilitates the recruitment of lysosomes to synapses, providing essential enzymes for immobilized antigen extraction and presentation.^212,213^ Moreover, centrosome-associated F-actin has been demonstrated to impede microtubule growth at the centrosome, a finding corroborated by in vitro reconstitution experiments utilizing purified centrosomes,^214^ suggesting that the clearance of synaptic F-actin alone is insufficient to polarize the centrosome to the IS. Rather, paxillin, a protein linked to the microtubule cytoskeleton, may contribute to actin‒microtubule crosstalk and facilitate repositioning of the centrosome to the IS.^215^

In addition to the above regulators, novel and unforeseen players have emerged in the regulation of centrosomal F-actin clearance and centrosome polarization during the assembly of the IS, with indications of the involvement of the ubiquitin‒proteasome system (UPS).^190^ The UPS, a pivotal degradation mechanism in eukaryotic cells responsible for the targeted proteolysis of cytoplasmic proteins that govern diverse cellular processes, comprises ubiquitin ligases that covalently add ubiquitin to substrates destined for degradation. Mounting evidence points to the role of the proteasome in maintaining centrosomal protein homeostasis.^216^ In B cells, the temporal and spatial coordination of centrosomal and synaptic F-actin depolymerization necessitates the repositioning of proteasomes from the centrosome to the IS.^190^ Proteomic analysis of centrosomes purified from activated T cells revealed local enrichment of proteasome components.^217^ While the reconfiguration of cortical F-actin has traditionally been deemed sufficient for centrosome polarization, the presence of a reticular F-actin network encompassing the centrosome (which must be eliminated during IS assembly) introduces an additional layer of intricacy to the regulation of this process. These distinctive networks collaborate to regulate the trafficking and localization of proteins within the T cell membrane, facilitating the targeted delivery of TCR recycling endosomes to the synapse.^218^ Therefore, exhausted TCRs that are internalized for recycling or lysosomal degradation are replenished with functional receptors at the synapse membrane.^218^ Alternatively, after endocytosis, TCRs are directed to multivesicular bodies and incorporated into intraluminal vesicles, which are subsequently released into the synapse cleft and engulfed by APCs.^92^ Emerging evidence suggests that centrosome-associated actin networks undergo dynamic cycling through repetitive polymerization and depolymerization events, contributing to polarized retrieval.^219^ F-actin clusters in recycling endosomes, aiding in the transport of recycled cargo, including TCRs, LFA-1, CD28, and the glucose transporter GLUT-1. These receptors accumulate at IS sites, participate in IS formation and maintenance, and reprogram the metabolic activity of activated T cells.^220^ Mitochondria are also mobilized to APC contact sites, where they bolster IS formation and TCR signaling by providing a localized source of ATP and modulating intracellular calcium concentrations.^219^

The actin cytoskeleton regulators also include galectins (such as Gal-1 and Gal-9) and tetraspanins (such as CD9, CD81, and CD82), which can interact with the actin cytoskeleton and actin-binding proteins and are therefore proposed as potential IS regulators.^221^ This notion is supported by the observation that CD81 binding to antibodies elicits marked morphological changes in T cells, along with localized F-actin accumulation during synapse formation.^222^ Similarly, CD82 is concentrated at sites of TCR activation and synapse formation, where it colocalizes with F-actin.^223^ These four transmembrane proteins likely influence the cytoskeleton through their intracellular interactions with proteins such as ezrin, radixin, and moesin (ERM proteins).^224^

In terms of subsequent functions, actin dynamics are believed to guide cytotoxicity or regulatory functions through cytokine production in T/NK cells as well as killing resistance in tumor cells. For example, the F-actin cross-linking protein FLNa has been shown to enhance multiple stages of NK cell cytotoxicity, including tethering to target cells, F-actin accumulation at synapses, and particle detachment, while suppressing the production of IFN-γ and perforin.^225^ Perforin release occurs at sites rich in actin-rich protrusions that extend from the synapse center and middle regions. These protrusions, which require regulators of the actin cytoskeleton, such as WASP and the Arp2/3 actin nucleation complex, are necessary for effective killing and mediating physical deformation of target cell surfaces during CTL‒target cell interactions.^103^ Given the central role of the cytoskeleton in IS, we specifically analyze its function in different contexts in the sections “Pre-synaptic cells” and “Post-synaptic cells”.

### Membrane reshaping in immunological synapse formation and disassembly

The membrane serves as the structural foundation of the IS and is crucial for both cells involved in the synapse. One of the major components of the cell membrane is cholesterol. Inhibiting cholesterol esterification in T cells via genetic ablation or pharmacological inhibition of ACAT1, a key cholesterol esterifying enzyme, promotes the proliferation of CD8^+^ T cells and enhances effector functions.^226^ This is due to the increased levels of cholesterol in the cytoplasmic membrane of CD8^+^ T cells, which leads to enhanced TCR clustering, signaling, and more efficient formation of the IS.^226^ In comparison, for tumor cells, T cell-mediated cytotoxicity is less effective against cancer cells with cholesterol-rich fluid membranes, while cancer cells with membrane hardening through cholesterol depletion lose resistance to CTLs and enhance the efficacy of adoptive T cell therapy in solid tumor models.^227^ The cell membrane at synapses is rugged and has nanoscale local curvatures. Membrane invaginations, driven by cytoskeletal forces, are prerequisites for T cell activation and unidirectional perforation of target cells.^84,191^ At the synapse, force-induced negative curvature membrane invaginations derived from actin-driven dynamin induce the clustering of granules that are transported to these invaginations. The highly concave degranulation pockets align against convex protrusions on the target cell membrane, allowing perforin to preferentially penetrate curved cancer cell membranes.^191^ Notably, although both CTLs and target cells expose perforin within the synapse, only the target cell membrane is disrupted, while CTLs are always spared. This is primarily achieved through two protective features of the CTL intracellular membrane at the synapse: on the one hand, the T cell membrane lipid bilayer is organized into an ordered structure consisting of cholesterol, sphingomyelin and (liquid-phase) phosphatidylcholine, which effectively reduces perforin binding;^228^ on the other hand, even if a small fraction of perforin enters the inner leaflet of the membrane, it is sequestered and inactivated by one of the receptors of Tim3, phosphatidylserine.^229,230^

**Fig. 3:**
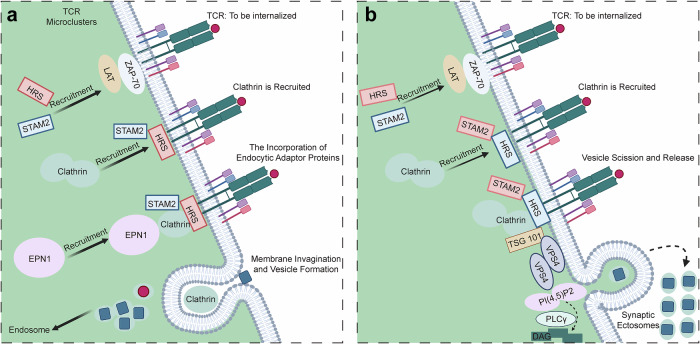
Endocytosis and ectocytosis of T cells. T cells downregulate surface TCR expression and downstream signaling upon activation primarily to avoid sustained overstimulation and timely disengagement from target cells either via endocytosis or exocytosis. These are two topologically opposite processes coordinated by the sequential recruitment of ecto- and endocytic adapters (HRS and STAM2). **a** EPN1 recruits clathrin to remaining TCR microclusters to enable trans-endocytosis of pMHC-TCR conjugates from the APCs through membrane invagination and vesicle formation. **b** TCR sheds from membranes via the coordination of clathrin recruitment and DAG signaling activation and then enters into outer bodies to mediate direct ectocytosis from the plasma membrane (called synaptic ectosomes). Image created with BioRender (https://biorender.com/)

The cell membrane of T cells is not static but undergoes a dynamic cycle of “gain–loss”. Typically, antigen-activated T cells acquire exogenous membrane proteins from APCs or tumor cells via trogocytosis, leading to rapid upregulation of TCR expression and metabolic reprogramming of cholesterol and fatty acid synthesis to support cell division and survival;^230^ however, it has also been reported that the loss of exogenous membrane proteins in T cells via trogocytosis during synapse formation increases their metabolism and clonal expansion^231^, underlying trogocytosis as a necessary process mediating cellular communication. Upon activation, T cells downregulate surface TCR expression and downstream signaling primarily to avoid sustained overstimulation and timely disengagement from target cells,^218^ a process involving the combined formation of caveolae and enzymatically driven vesicles that results in the loss of membrane-bound TCRs and associated proteins and lipids, ultimately leading to TCR downregulation.^232^ TCR shedding occurs via two independent pathways, either via ectocytosis released as EVs from activated T cells, together with exosomes, microvesicles, and LGs, or via endocytosis (Fig. 3).^232,233^ TCRs are highly enriched in microvilli tips, and upon T cell activation, microvilli are fragmented into nanoscale membrane particles enriched in TCRs called TMPs.^232^ The endosomal sorting complex required for transport (ESCRT) system is a protein transport machine. After clathrins are recruited to TCR microclusters and TMPs by ESCRT-0 proteins (HRS and STAM2), TCRs are released from the plasma membrane directly through exocytosis.^231,232^ Released microvesicles or membrane particles can trigger signaling in antigen-bearing B cells and DCs, indicating that these nanoscale membrane particles can transfer T-cell-derived signals to their cognate APCs.^92^ Furthermore, T cells can release a fourth class of signaling entities, trans-synaptic vesicles (tSVs), to mediate bidirectional communication. The integration of juxtacrine signals, such as CD40 and antigens, results in the adaptive tailoring and release of tSVs, which differ in size, yield, and immune receptor cargo compared with steadily released EVs.^234^ Subsequently, the endocytic adapter protein epsin 1 (EPN1) recruits clathrins to the remaining TCR microclusters to achieve retrograde endocytosis of TCR‒pMHC complexes from APCs. The coordination of these two processes by sequential recruitment of endocytic and exocytic adapters controls bidirectional membrane exchange at the IS, providing a scaffold for direct bidirectional communication between T cells and APCs.^232^ It was previously proposed that endocytosis rather than ectocytosis dominated the process of TCR downregulation.^232^ However, recent studies have challenged this notion. Researchers, using high-resolution three-dimensional (3D) imaging, tracked the fate of TCRs at different stages of IS formation and reported that activated TCRs are shed into detergent-resistant outer bodies enriched with diacylglycerol (DAG) at the IS rather than internalized via endocytosis, terminating TCR signaling and enabling CTL detachment from target cells for continued killing.^233^

The outward budding process of ectocytosis requires the involvement of DAG, a metabolic product generated by plasma membrane phospholipid remodeling following TCR activation, which plays a crucial role in the actin-mediated mechanosensing network at the IS.^235^ In brief, TCR engagement activates PLCγ, cleaving PI(4,5)P2 (a lipid known to interact with actin regulatory proteins) to generate DAGs, reducing actin recruitment and generating DAG-enriched membrane domains.^109,236^ Moreover, changes in phosphoinositide during the formation of synapses alter the membrane charge, excluding phosphatidylinositol 4-phosphate 5-kinase type I (PIP5K), which is required for replenishing PI(4,5)P2, further increasing the consumption of transmembrane actin during secretion.^235^ Consequently, forced expression of PIP5K at the synapse impairs granule secretion and cytotoxic properties in CTLs.^228,235^ This actin loss model can also explain the flattening of membranes upon CTL–target contact, as the driving force of lamellipodial extension by the Arp2/3 branched actin network dissipates rapidly from the projecting tips. In B cells, diacylglycerol kinase zeta (DGKz) similarly promotes LFA-1-mediated adhesion and F-actin accumulation at the IS by generating phosphatidic acid (PA) from DAG. DGKz deficiency or depletion of DAG impairs the force exerted on the IS by B cells, resulting in the reduced antigen extraction required for presentation to T cells.^237^

Other molecules that regulate membrane exchange and dynamic remodeling at the IS also include Ca^2+^, Mg^2+^, and Zn^2+^, which are collectively referred to as the second messengers of the IS. Ca^2+^ signaling plays a crucial regulatory role in cellular biology, and calcium is a nonsynthetic and highly diffusible early second messenger in T cells that contributes to the initial steps of IS formation and is crucial for the function of immune cells, including cell–cell adhesion,^238^ the regulation of signaling kinases,^239^ actin cytoskeleton remodeling,^240^ and LG secretion.^241^ In CTLs, the dynamics of Ca^2+^ signaling are influenced by the stability of CTL interactions with APCs, thus finely regulating effector functions. Interaction with APCs carrying antigenic pMHC leads to a rapid increase in cytoplasmic Ca^2+^, and when there is a lack of co-stimulation during high-affinity antigen peptide activation, T cells have transient and weak Ca^2+^ spikes upon brief contact with APCs.^242^ Prolonged or sequential IS formation requires extended interactions with antigen stimulation. Combined with sustained intracellular Ca^2+^ signaling, this leads to the nuclear translocation of the activated T-cell’s nuclear factor of activated T cells (NF-AT) and increased cytokine gene expression.^243^ For Ca^2+^ ions to function as on/off switches for signaling molecules, their concentrations within cellular microstructural domains must change over time. For example, Ca^2+^ that reaches the cytosol upon cell stimulation must subsequently exit the extracellular space or be sequestered within the ER as the signaling cascade is interrupted. However, owing to their charge, ions cannot freely diffuse on the lipid bilayer of biological membranes but require transmembrane channels and transporters such as Ca2^+^-Release Activated Ca2^+^ (CRAC) channels to regulate Ca^2+^ concentrations within the cytosol and organelles.^219^ When the calcium flux increases through the CRAC channel, actin is depolymerized, and the concentration of F-actin in the central region of the IS is reduced (possibly through Ca^2+^-sensitive actin-severing proteins, e.g., gelsolin), thus limiting actin polymerization to more distal regions.^244^ Moreover, mitochondria undergo translocation to the IS, enabling ER‒mitochondrial contacts to occur,^197^ resulting in defects in the downstream activation of classical signaling events, such as myosin II phosphorylation.^245^

The orchestration of actin polymerization extends beyond the sole regulation by Ca^2+^ to encompass the influence of Mg^2+^. Insights gleaned from cryo-electron microscopy (cryo-EM) revealed a meticulous rearrangement of water molecules within the nucleotide-binding enclave of actin upon the initiation of polymerization, thereby catalyzing a singular molecule for an ATP nucleophilic onslaught.^244^ Subsequent to the hydrolysis of ATP and the liberation of Pi, minute alterations within the nucleotide-binding site are discerned by amino acids, magnified, and conveyed to the outer edges of the filament. In this intricate process, Mg^2+^ and Ca^2+^ form a complex coordination framework with ADP and water molecules. However, the distinct spatial arrangements of water molecules in the nucleotide-binding pockets of these two F-actin variants elucidate the relatively slower polymerization kinetics of Ca^2+^-actin than those of Mg^2+^-actin.^244^ Notably, magnesium ions have also been implicated in the dynamic configuration of LFA-1 on the surface of CD8^+^ T cells, thereby instigating increased calcium flow, propagating signaling cascades, fostering metabolic reconfiguration, facilitating IS assembly, and ultimately culminating in targeted cytotoxicity.^246^ This phenomenon may elucidate the observed clinical correlation between diminished serum magnesium levels and increased disease progression, along with decreased overall survival rates in individuals receiving CAR-T-cell therapy and immune checkpoint antibody interventions. Fundamentally, Mg^2+^ has the capacity to interrogate the microenvironmental landscape, acting as a pivotal determinant in the orchestration of extrinsic-to-intrinsic signal transduction pathways, conceptually interweaving notions of co-stimulation and nutrient perception, and underscoring the therapeutic potential of the Mg^2+^-LFA-1 axis as a modifiable biological entity.^246^

Finally, emerging discoveries from cryo-EM and imaging mass spectrometry also suggest the impactful role of zinc in instigating the formation of the IS.^247^ Under homeostatic conditions, zinc promotes the assembly of soluble KIRs into filamentous arrays, enabling their interaction with HLA-C to form inhibitory ISs that restrain the activation signals impinging upon human NK cells.^247^ In the absence of zinc, KIR has the ability to maintain a distinct conformation, thereby relinquishing its inhibitory sway over NK cell activation cues.^247^

### Integrin signaling in immunological synapse formation and disassembly

Immune cells are adept at migrating, facilitating prompt recruitment yet necessitating a mechanism for “stasis” to enable intercellular communication.^248^ The integrin ligand‒receptor pair serves as a fundamental axis for cellular adhesion and the formation of the IS, typically acting as a bidirectional transducer of signals between the intracellular and extracellular environments. Notably, integrins do not operate in isolation; antigen receptors, such as the TCR, often collaborate with integrins as integral components of the adhesive and stimulatory interface.^249^ Upon encountering target cells, the TCR/BCR structures engage corresponding ligands, initiating an “inside-out” signaling cascade within T/B cells. This process transforms integrins from inactive to active conformations, thereby enhancing T cell adhesion.^250,251^ Subsequently, “outside-in” co-stimulatory signaling is activated, supplanting “inside-out” signals, reducing the threshold for antigen-dependent lymphocyte activation, and further stabilizing the interaction between T cells and APCs, thereby promoting T cell activation.^250,251^ Following activation, the dissociation of T/B cells is also contingent upon conformational changes in integrins, as their affinity for ligands diminishes and they undergo internalization, ultimately detaching from the plasma membrane.^82^

The most renowned integrin receptor in T cells, LFA-1, predominantly accumulates at the central membrane of the IS, fine-tuning cellular adhesion. Following the activation of “inside-out” signals, F-actin within the cell forms a “reverse flow band” and promotes the conformational transition of LFA-1, which facilitates the rearrangement of LFA-1 on the T-cell membrane and positions.^252^ The use of F-actin stabilizers such as jasplakinolide and the myosin II inhibitor blebbistatin or the knockdown of Cofilin1 (CFL1, an actin-binding protein that causes the depolymerization of F-actin) to inhibit F-actin depolymerization can disrupt the formation of this “F-actin reverse flow band”, thereby diminishing the accumulation of high-affinity LFA-1 at the IS.^252^ Upon the transmission of “inside-out” signals to ICAM1 on the target cell surface, a similar rearrangement of the actin network within the ICAM domain of the target cell can be induced.^174^ Under normal circumstances, the migration and aggregation of ICAM-1 on the cell membrane are regulated and constrained by moesin and α-actinin-1; conversely, under pathological conditions, DC surface ICAM-1 exhibits increased migratory rates, thereby compromising the formation of antigen‒antibody complexes and the priming of T cells.^174^ The migration of ICAM-1 is also regulated by talin, which recruits vinculin and F-actin to the integrin cytoplasmic tails at the T-APC plasma membrane contact sites, thereby stabilizing the interaction.^253^ After LFA-1 and ICAM-1 interact stably, phosphorylation of the β2 integrin chain is triggered, initiating co-stimulatory signaling events in T cells and leading to cytoskeletal rearrangement and nuclear activation.^254^

Throughout the entire process of bidirectional signal transmission, the TCR/BCR complex plays a pivotal role. To analyze the crosstalk between TCRs and LFA-1, Tabdanov et al. imaged T cells on micro-patterned surfaces containing stimulatory anti-TCR antibodies and ICAM-1 in isolated domains.^255^ They reported that the TCR stimulation zone colocalized with the Arp2/3 complex, which induced the formation of branched F-actin arrays, and that Arp2/3 was essential for the TCR-induced response. In contrast, the ICAM-1 domains are characterized by aggregated FHOD1 proteins (a formin needed for the organization of lamellar actin, integrin adhesion maturation and cell spreading), which assemble linear F-actin bundles.^255,256^ Interestingly, the inhibition of Arp2/3 also dampened actin polymerization within the ICAM-1 domains, despite the minimal accumulation of Arp2/3 in these areas.^257^ This finding suggests that Arp2/3-dependent actin polymerization downstream of the TCR is instrumental in “priming” the formation of integrin-based structures, which concurs with previous studies suggesting the role of F-actin in integrin-mediated rapid stable adhesion.^258^ Moreover, the presence of TCR contributes to the stabilization of “microvillar” structures on the T cell surface, which are dependent on the remodeling of the actin cytoskeleton and facilitate the scanning of the APC surface by T cells, thereby enabling TCR-dependent activation.^198,259^

The development of antibodies targeting integrin molecules has been realized, yet these anti-adhesion immunotherapies have not achieved the same level of success as immune checkpoint inhibitors. On the one hand, cancer cells may evade antibodies directed at specific integrins by expressing alternative integrins or utilizing other adhesive molecules during metastasis.^260^ For example, in addition to LFA-1, CD103 can also bind to E-cadherin at the IS formed with target cells, thereby enhancing the cytotoxic effects of CD8^+^ T cells against tumor cells.^261^ On the other hand, these anti-adhesion therapies, such as anti-integrin antibodies, may nonspecifically target ECM proteins such as fibronectin, tenascin, or vitronectin and affect the quality of the IS established within the tumor microenvironment, thus impeding immune protection.^262^ Hence, deepening our understanding of the relationship between integrin molecules and the IS is imperative to provide a theoretical foundation for future integrin-based therapies.

### Force signaling in immunological synapse formation and disassembly

The classic model of mechanotransduction refers to force-induced conformational changes in the extracellular domain of receptors, which are transmitted through the cell membrane, leading to structural changes in the cytoplasmic domain and ultimately triggering downstream signaling events.^192^ Over the past decade, it has become increasingly evident that T/B cells are sensitive to the physical properties of antigens. For example, the TCR can convert mechanical forces exerted and sensed during pMHC complex binding into biochemical signals,^263^ implying that it is a mechanosensor.

**Fig. 4:**
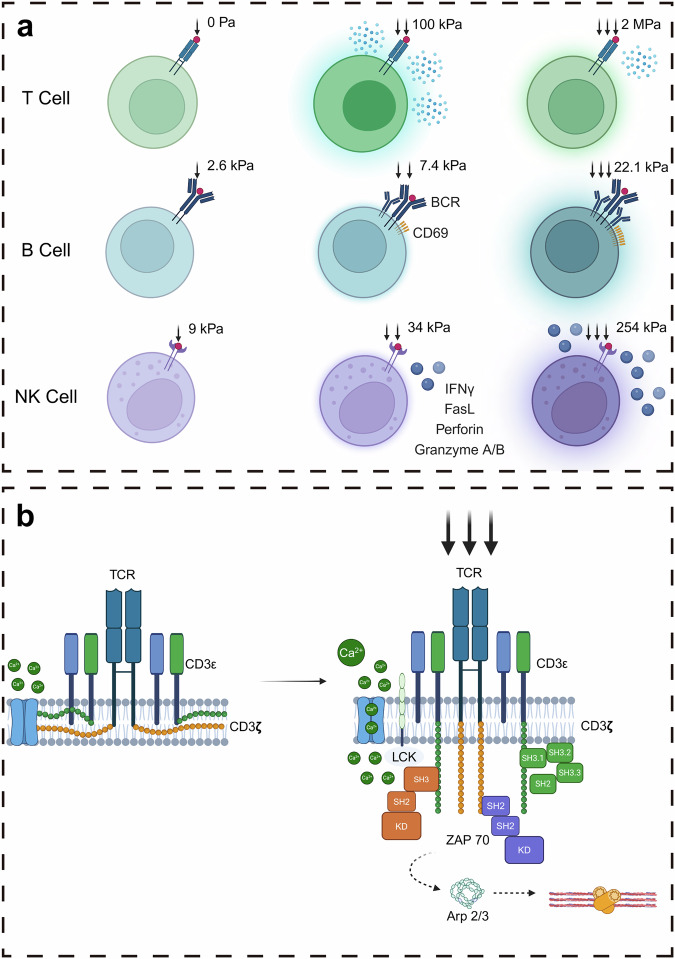
Force signaling transduction of lymphocytes. **a** Activation of three lymphocyte subtypes under various external stimuli; **b** Conformational changes in the intracellular domain of the TCR upon mechanical stimuli. In the resting state, CD3ε and CD3ζ are inserted into the membrane through electrostatic interactions, preventing their phosphorylation by Lck. In the open state, ITAM fully dissociates from the plasma membrane. The Tyr residues within ITAM are phosphorylated by Lck, recruiting downstream signaling molecules such as Zap70. The PRS region recruits Nck, leading to cytoskeletal remodeling to accommodate T cell activation. Image created with BioRender (https://biorender.com/)

The first clue regarding this phenomenon came from an in vitro experiment in which stimulating ligands were applied to T cells on surfaces of varying hardness.^264^ Using polyacrylamide hydrogel substrates coated with anti-TCR antibodies, it was observed that stiffer surfaces (100–200 kPa) had greater stimulatory effects on mouse CD4^+^ T cells than softer surfaces did, leading to higher levels of intracellular signaling and subsequent cytokine secretion.^264^ However, a subsequent study revealed that surfaces with a stiffness of ~100 kPa created with polydimethylsiloxane elastomer had a significantly greater stimulatory effect on human CD4^+^ T cells than harder ( ~ 2 MPa) substrates.^265^ Researchers thus propose that TCR responsiveness to stimulus hardness may exhibit a bell-shaped response, peaking at ~100 kPa. However, it is also possible that polydimethylsiloxane and hydrogel-mediated T cell activation differ depending on the material or that the mouse T cells used in the first study differ from the human T cells used in the subsequent study. Therefore, researchers have employed another informative experimental design in which forces are directly inhibited by T cells via long tethers (lengths of membrane tethers indicating receptor–ligand affinity) to investigate whether individual ligand engagements are sufficient to trigger signal transduction in the absence of tension on the TCR–ligand bond.^266^ Activation was not detected when T cells were stimulated under static conditions via this tether, but when external forces were applied, calcium flux-based activation signaling was detected, indicating the need for forces in the piconewton range to trigger TCR signaling.^267,268^ Similarly, stimulation with hard (142 kPa) substrates, compared with soft (1 kPa) planar substrates, leads to more potent secretion of granzymes A and B, FasL (also known as FasLG), perforin, and IFN-γ in human NK cells.^269^ To create alternative spherical targets with specific stiffness, sodium alginate was used to synthesize beads of cellular size categorized as soft (9 kPa), medium (34 kPa), or hard (254 kPa) and coated with antibodies against the activating receptor NKp30 (also known as NCR3) and the integrin LFA-1.^269^ NK cells demonstrated enhanced degranulation in response to rigid beads, whereas the polarization of centrosomes and LGs was impaired against soft targets, culminating in the establishment of unstable ISs (Fig. 4).^269^

Similar substrate hardness experiments have also been conducted on B cells: stiffer antigen hydrogels and polydimethylsiloxane surfaces can elicit stronger early B cell receptor (BCR) signaling and effector responses, whereas softer polydimethylsiloxane surfaces seem to be optimal for prolonged proliferation responses.^270^ Similar to studies on T cells, the underlying mechanisms for these differential effects are still unclear. By coupling antigens to DNA-based tension gauge tether probes (using membrane-specific probes measuring the extent of membrane tension), B cells can be mechanically pulled on their ligands at the single-molecule level, and importantly, continuous force application is required for optimal signal transduction.^93^ B cells additionally employ the process of BCR internalization to capture antigens, enabling their presentation to helper T cells and subsequent signal transmission for expansion and differentiation. Recent studies have shown that myosin-dependent forces are crucial for this process.^271,272^ Through the use of imaging techniques on fixed supported membranes incorporating fluorescently labeled antigens (characterized by more physiologically relevant viscoelastic properties than planar lipid bilayers), the process of antigen uptake by B cells can be delineated into a sequence of discrete steps.^271^ Initially, the target membrane harboring the antigen is pulled inward into the IS via myosin-dependent forces. A cage-like protein complex subsequently forms around the invaginated membrane, marking the completion of the internalization process. The majority of invaginations rupture prior to the assembly of the lattice proteins; however, those containing large antigen clusters, which potentially experience more BCR binding events, retain their structure sufficiently (for approximately 20 s) to facilitate productive endocytosis.^271^ These findings suggest that only high-affinity antigen particles (i.e., large antigen clusters) can maintain their structure under the influence of cytoskeletal forces, allowing B cells to preferentially select high-quality antigens. Consistent with this hypothesis, blocking myosin-II has been found to impair the ability of B cells to differentiate between high-affinity and low-affinity antigens during phagocytosis.^272^ Thus, similar to the TCR, applied forces significantly influence the ability of B cells to recognize and respond to antigenic stimuli.

The role of forces in immune cells has been extensively investigated by immunologists, and various dynamic models have shed light on their specific functions. The kinetic segregation model explains how the size-based partitioning of cell surface proteins can initiate signaling at the IS upon TCR/BCR binding to specific antigens.^273^ The kinetic proofreading model suggests that T cells can scan a large number of MHC molecules carrying self-peptides, as the initial TCR engagement is insufficient for productive T cell activation, and short-lived non-specific TCR-pMHC bonds dissociate before activation.^274^ The serial triggering model addresses why only a small fraction of specific pMHC molecules activate the TCR, highlighting the importance of stable interactions lasting shorter than the half-life of triggering the next round of activation.^275^ These models highlight the crucial role of mechanical forces exerted by the actin cytoskeleton in inducing TCR–pMHC catch–bonding and disrupting this binding before the next activation step.^276^ Additionally, the dynamic architecture of microvilli on T cell surfaces contributes to TCR kinetics, allowing for close contact zones even without pMHC stimuli and facilitating rapid scanning of antigen-presenting cells for rare stimulating pMHCs.^119,205,277,278^ Notably, microvilli motion slows during catch-bond interactions, potentially enhancing antigen recognition.^277^ Although the antigen recognition mechanism depends on the T cell actin cytoskeleton, external forces to the TCR-pMHC bond can also activate the TCR, even in the absence of actin depolymerization, providing insights into the intricate interplay between mechanical forces and immune cell responses.^119,278^ The forces exerted in conjunction with the actin cortex generate rapid, long-range membrane tension propagation, defined as resistance to membrane deformation, whereas forces applied solely on the cell membrane cannot propagate through membrane tension.^279^ In cellular systems, membrane tension is regarded as a composite of in-plane tension and adhesion between the membrane and the underlying actin cytoskeletal network. This long-range propagation of tension facilitates communication between protrusions, thereby facilitating the establishment of a dominant axis for competing with cellular motility.^279^ Spontaneous fluctuations of T lymphocyte membranes, as well as the formation/retraction of filopodia and other cellular protrusions, can generate forces that aid in sensing the biomechanical microenvironment.^280^ Lymphocytes likely possess intricate mechanisms to safeguard their surface receptors against potentially impactful forces stemming from tissue motion, shear flow, and other extraneous origins.^100^ An exemplary illustration lies in the IS, wherein an ample F-actin belt enfolds its periphery, potentially shielding the interface from the influence of surrounding external forces.^100^ Manipulating F-actin assembly or depolymerizing existing filaments significantly reduces forces when applied before or early in IS formation, as observed by the substantial reduction in force with myosin inhibitors;^281^ however, blocking myosin activity in mature ISs (approximately 15 min after contact formation) has little effect on force maintenance over time.^66^

Importantly, cytoskeletal organization and forces appear to vary with different types of immune cells and their functions, but it is difficult to determine causality. Distinct levels of mechanical activity were observed among exhausted T cells (Tex), effector memory T cells (Tem), effector T cells (Teff), and Tex as well as opposing mechanotypes between bone marrow-derived macrophages (BMDMs) and T cells.^62^ CTLs, in particular, exhibit more intricate and robust distortions than other T cells do, with Teffs displaying higher protrusion numbers and peak relief convexity.^62^ Previous reports have shown elevated peak indentation concavity and peak relief convexity at the IS relative to non-synaptic membrane regions.^282^ Additionally, cytoskeletal proteins such as WASP, WAVE2, and talin have crucial impacts on the curvature of the IS.^282^ Consequently, the proposition arises that cytoskeletal forces regulate downstream synapse formation and T cell function.

At present, a comprehensive understanding of the intricate mechanisms underlying TCR/BCR mechanotransduction signaling and its precise regulatory role in specific physiological phenomena remains elusive. Nonetheless, certain inferences can be deduced from inconsistent findings. Forces applied to the extracellular TCRαβ chains can result in CD3 phosphorylation, subsequently exposing CD3 ITAMs in the T cell cytoplasm to allow increased binding of ZAP-70, leading to propagation of downstream signaling, with conformational changes in CD3 observed during this process.^98,283,284^ Moreover, early tyrosine phosphorylation events downstream of TCR engagement occur at sites of maximal forces, which are also sites of LG secretion.^66,285^ Nanoscale actomyosin dynamics mediated by the Arp2/3 complex and myosin IIA can generate the mechanical forces that promote degranulation.^105,286^ Indeed, tension on LFA-1 has also been reported to impact the dynamics of the T cell actin network.^287^ Further investigations via atomic force microscopy (AFM) have demonstrated that the affinity between the α5β1 integrin and its fibronectin ligand increases under applied forces, with a threshold of at least 30 pN.^288^ Notably, integrins, particularly the αLβ2 isoform LFA-1, play a pivotal role in binding ligands within spatial clue-containing secretory synapses. This interaction facilitates the recruitment of perforin and granzyme-containing LGs and triggers their fusion with the plasma membrane, thus releasing their contents.^288^ LFA-1 experiences tension within the secretory domain, and disrupting these forces by depleting the linker protein talin eliminates cytotoxicity.^288^ In addition, experiments utilizing functionalized stiff beads coated with antibodies against the NK activation receptors NKp30 and LFA-1 have shown remarkable efficacy in triggering degranulation in NK cells. Conversely, softer beads fail to induce MTOC and LG polarization in NK cells, leading only to the formation of unstable IS structures.^269,289^ These findings suggest that lymphocytes employ integrin-dependent mechanical checkpoints to increase their cytotoxic capabilities and fidelity.

By spectral decomposition of the force patterns exerted by each cell type, the induction of cytotoxicity was correlated with compressive strength, local prominence, and complex asymmetric interface topology.^62^ These features were further validated as driving factors for cytotoxicity through genetic disruption of cytoskeletal regulators, direct imaging of synaptic secretion events, and computer analysis of interface distortions.^62^ Single-cell imaging experiments revealed that PTEN depletion in CTLs leads to a significant increase in the rate of perforin pore formation in target cells.^66^ On the other hand, the knockdown of DOCK-2 in T cells results in the formation of smaller synapses, reduced forces, and diminished cytotoxicity.^66^ This phenotype cannot be attributed to increased LG release or perforin expression but is likely due to mechanical enhancement of perforin function at the IS, as DOCK-2- and PTEN-deficient cells do not exhibit defects in calcium flux, centrosome repositioning, or LG release (as measured by surface LAMP-1 expression), which is consistent with previous observations that membrane tension can modulate pore-forming peptide activity.^290^ These data collectively indicate that the forces exerted by the T cell actin cytoskeleton at the IS contribute to the enhancement of cytotoxicity through the augmentation of tension within the target cell plasma membrane, thereby facilitating the formation of perforin pores and promoting the delivery of LG and subsequent cell death.^66^ An in-depth investigation into the nexus between cellular stiffness and perforin efficacy was subsequently conducted via the deployment of polyacrylamide hydrogels.^291^ Adherent cells on stiff hydrogels adopt a star-shaped configuration with increased surface and membrane tension and contain numerous actin stress fibers, whereas cells on soft hydrogels exhibit a more collapsed morphology and lack stress fibers.^291^ Crucially, tumor cells seeded on inflexible matrices demonstrated heightened responsiveness to both purified perforin and CTL killing than their counterparts seeded on compliant substrates did, indicating the essential role of the tensile environment in modulating perforin efficacy within the IS.^66^

Despite the significant role of mechanical force in immune cells, the scenario is significantly nuanced when considering intercellular communication in both in vitro and in vivo settings. Initially, T lymphocytes engage in antigen-independent pushing interactions with neighboring cells, a process that is succeeded by the recognition of cognate antigens, after which T cells exert pulling forces to draw APCs into proximity to establish secure connections characterized by distinct kinetics and delineated by discrete temporal intervals.^292^ While the forces applied to the TCR originate from actomyosin-dependent pushing and pulling exerted by T cells against APC surfaces,^76,285,293^ they are also dependent on the biophysical properties of the APCs, particularly their cortical stiffness, which is primarily controlled by the cytoskeleton. We previously introduced several studies exploring the impact of forces on TCR activation in vitro; however, the measurements of forces applied to T cells mentioned above were derived from studies involving interactions with extremely stiff surfaces in the gigapascal range. In contrast, cells in vivo are much softer, with cortical stiffness values ranging from 5 Pa to 40 kPa, and APCs exhibit lower stiffness values at the lower end of this range.^294^

The biophysical properties of the APC cortex can regulate TCR deformation and signaling, with DC differentiation optimized for triggering T cells and cortex hardness increased 2–3-fold (actomyosin-dependent). While this seems to be a moderate increase (from 2 kPa in immature DCs to 4–6 kPa in mature DCs), T cells stimulated on the surface with 2 kPa show almost no response, whereas T cells stimulated on the surfaces with 4–8 kPa effectively proliferate, which is perceived by T cells as a co-stimulatory signal.^85^ Cortical stiffening enhances mechanical stimulation of the TCR complex, resulting in increased downstream signaling and a decrease in the antigen threshold required to activate T cells.^64,267^ Like in T cells, the Arp2/3 complex and formin-mediated actin polymerization contribute to increased cortical stiffness in mature DCs.^85^

## Immunological synapses in health

The immunological synapse is a major core weapon of the immune system, and it undoubtedly plays a crucial role in basic functions of the immune system, such as immune surveillance, immune defense, and immune regulation. In addition, the IS also possesses some surprising additional functions; for example, immature immunological synapses are vital for maintaining immune tolerance,^13,15^ and the phagocytic synapses of microglia play a significant role in neural development and homeostasis of the central nervous system.^16,295^ Understanding these physiological functions will help in the development of more comprehensive clinical treatment strategies.

### Immune surveillance and host defense

Immune surveillance refers to the body’s ability to detect and eliminate non-self components that appear within it, such as tumor cells. Adaptive immune cells (αβ T cells, γδ T cells, and B cells) and innate immune cells (NK cells and macrophages) play crucial roles in this process. Cytotoxic mediators, such as IFN-γ, perforin, and granzymes, are major weapons used by T cells and NK cells to eliminate tumors. Multiple studies have shown that T cells and NK cells must form stimulatory synapses and be activated before they can secrete large amounts of these mediators.^10,43,180^ Additionally, another function of the IS is to confine these cytokines to a small space, thereby maximizing their local concentration to increase their efficiency in eliminating tumors. Recently, a special form of synaptic secretion from T cells^296^ and NK cells^297^, in which these cytotoxic multi-protein complexes are called supramolecular attack particles (SMAPs), was reported. Researchers have subsequently proposed that CTLs use SCG fusion (releasing soluble Granzyme B) for instant killing and delivery of latent SMAPs for delayed killing of refractory targets.^102^

The importance of the IS in tumor immune surveillance is also reflected in its role as the direct interface where immune cells “kill” tumor cells. Cytotoxic T cells or NK cells can form lytic ISs with tumor target cells via mechanisms such as MTOC polarization, cytoskeletal remodeling, and changes in the cell membrane structure to exert sufficient “pore-forming force” on the target cell membrane, thereby disrupting it and initiating its apoptotic program to eliminate the tumor. Researchers have already discovered the central role of the IS in tumor immune surveillance in various cancers, and the immune escape caused by IS dysfunction will be discussed in subsequent chapters.

The IS also assists the body in building a defense system to prevent the invasion of external pathogens and eliminate those that have already invaded.^8,9^ Monocytes are among the main innate immune cells that mediate the body’s anti-infectious immunity, among which macrophages can sense granular β-glucan and phagocytose target pathogens by forming “phagocytic synapses“^298^ while simultaneously initiating direct cellular antibacterial responses, including the activation of reactive oxygen species production and the stimulation of inflammatory responses.^8^ Surprisingly, T lymphocytes can form ISs with infected DCs, take up bacteria, and produce high levels of pro-inflammatory cytokines within the first few hours of infection, killing bacteria in a manner similar to innate immunity.^299^ Adaptive immunity is the main way in which T lymphocytes fight infection, which requires the formation of an IS with antigen-presenting cells such as DCs that have phagocytosed and processed pathogens before being activated.^300^ Recent research has shown that the IS is essential for the existence of the CD4^+^ T-DC-CD8^+^ T cell anti-infectious chain.^9,301,302^ The antigen homologous DC‒T-cell synapse interaction first drives the activation of CD4^+^ T cells and then guides DC.^301^ After receiving help from CD4^+^ T cells, psDCs are licensed to generate CD8^+^ T cell responses, ultimately protecting mice from infection in a CD8^+^ T cell-dependent manner.^9^ The adoptive transfer of psDCs enhances the pathogen-specific CD8^+^ T response, while the depletion of psDCs in vivo eliminates the antigen-specific CD8^+^ T cell response during the immune response,^9^ emphasizing the core role of IS mediated immune licensing in host defense against pathogens.

### Immune regulation

The immune regulatory mechanism is crucial for maintaining the stability of the internal environment of the body. If immune regulatory function is abnormal and the body’s own components are subjected to strong immune attack, leading to cell destruction and loss of function, autoimmune diseases can occur.^303^ If the body cannot produce an appropriate response to infection by external pathogenic microorganisms (a low response can lead to severe infection, whereas an excessive response can cause allergic reactions), it can also have harmful effects on the body.^304^ The IS is a key structure for immune cells to perform immune regulation. T cells have potent compartmentalization mechanisms that can strictly control the assembly of the IS in time and space through compartmentalized cyclic AMP production, ensuring that even when cAMP is produced at supraphysiological levels, it is confined to the vicinity of its production, preventing the expansion of inflammation.^305^

The IS regulates immune activity through two distinct mechanisms: contact-dependent regulation and secretory regulation. Both T cells and NK cells have been demonstrated to form stimulatory or inhibitory synapses directly with APCs and tumor target cells, modulating immune activity through their coordinated dual actions to combat tumors^11,12^ or pathogens^9^ while maintaining organismal homeostasis.^71,135^ Furthermore, the dissociation of the IS is tightly regulated,^233,306^ effectively braking activated immune responses and preventing autoimmunity.^304^

The secretory regulatory function of the IS represents an intriguing phenomenon that has attracted increasing attention in recent years.^187,188,307–309^ This process involves immune cells secreting synaptic products as informational messengers to adjacent^307^ or distant^308^ immune cells, thereby modulating recipient cell functions. These synaptic products may include membrane components of the IS itself, such as the recently reported “T Cell Immunological Synaptosomes”,^187,188^ which consists of synaptic ectosomes combined with vesiculated TMPs—structures previously considered merely structural scaffolds for TCR clustering. More common are EVs and particles secreted during synapse formation. These compounds can be released from activated immune cells to increase the frequency of IS in recipient cells through proximity-independent mechanisms, enabling local and systemic immune regulation in inflammation, autoimmunity, organ fibrosis, cancer, and infections.^308^ For example, tumor proteins internalized by DCs in lymph nodes are transferred to distinct vesicles that can be transmitted to adjacent DCs during IS formation, conferring T cell activation capacity.^307^ These vesicles may also carry bioactive molecules such as microRNAs, transferring them from T lymphocytes to antigen-presenting cells (e.g., B cells) through the IS to activate adaptive immunity.^310^ Notably, certain microvesicles at the IS may contain combinations of ligands and receptors that simultaneously interact with cell-surface molecules on recipient cells, mediating immune activation processes analogous to contact-dependent regulation.^92,309^

In addition to these established regulatory mechanisms, recent studies have revealed a novel protective function: safeguarding immune cells against senescence.^311^ While the conventional understanding holds that synaptic stimulation activates telomerase as part of immune responses in T cells,^312^ repeated synaptic interactions progressively diminish telomerase activation,^313^ leading to T cell senescence characteristics, compromised immune memory, and increased susceptibility to infections and cancer. However, this paradigm fails to explain the long-term maintenance of T memory cells. Compositional analysis of vesicles transferred between APCs and T cells provides new insights: APCs contacting naive and central memory T cells can degrade shelterin complexes and transfer truncated telomeres to T cells via EVs at the IS, conferring long-term protection against senescence.^311^

### Neurodevelopment and memory loss

Microglia are recognized as resident macrophages of the central nervous system.^295^ They phagocytose and clear cellular debris through synaptic engulfment, such as fragments derived from axosome shedding^314^—membrane-bound remnants generated during neurodevelopment. Furthermore, they directly mediate “synaptic pruning” to facilitate postnatal brain development and synaptic maturation,^16^ although the precise mechanisms of this pruning remain controversial.^295^ Studies utilizing light sheet fluorescence microscopy to track microglia‒synapse interactions in developing organotypic hippocampal cultures have revealed dynamic interaction patterns, including selective partial phagocytosis or trogocytosis (from the Greek “trogo” meaning “nibble”) of presynaptic structures.^315^ Complementary in vivo models have demonstrated microglia-mediated axonal remodeling via trogocytosis during neural circuit development,^316^ corroborating these findings. This process is regulated by the complement system, which simultaneously suppresses synaptic phagocytosis and axonal pruning,^316^ establishing a balance between pro-phagocytic “eat-me” signals (e.g., complement component C1q) and anti-phagocytic “don’t-eat-me” signals (e.g., transmembrane glycoprotein CD47) to modulate microglial synaptic engulfment.^317^ Complement-dependent synaptic elimination by microglia is crucial for remote memory forgetting,^318^ and repeated low-dose LPS induction in adult mice has been shown to impair this function and enhance memory retention.^319^ Collectively, these studies underscore the essential role of microglial phagocytosis in normal neurodevelopment and physiological processes. However, research specifically investigating the functional significance of synapse phagocytosis itself remains limited, hindering the establishment of more comprehensive mechanistic correlations.

### Immune tolerance and thymic development

Mature ISs significantly regulate immune homeostasis, while immature synapses also possess intrinsic regulatory mechanisms to mediate immune tolerance or immune privilege.^13–15^ As early as the 2000s, studies revealed that immature ISs formed by maternal decidual NK cells during early pregnancy protect fetal trophoblasts from immune attack, representing the first reported instance of immature synapses mediating immune privilege.^13^ Mechanistically, complement component C3 can be hydrolyzed into two competing fragments: C3d, which facilitates synapse formation, and iC3b, which inhibits synapse formation. These fragments dynamically compete for binding to the CR3 receptor on target cells, determining whether immune cells respond to cognate antigens.^14^ Host cells utilize regulators of complement activation (RCAs) to coat themselves with iC3b, thereby mediating self-tolerance by preventing synapse formation.^14^ Regulatory T cells (Tregs) constitute another key subset that mediates immune tolerance. They suppress effector T cell responses to cognate antigens by forming ISs with them^320^ or indirectly regulate peripheral homeostasis by establishing long-lasting MHC-II-independent adhesive contacts with DCs to decrease their costimulatory capacity.^321^ Further studies demonstrated that upon interacting with cognate DCs, antigen-specific Tregs induce the removal of peptide-MHC class II (pMHCII) complexes from DC surfaces, selectively inhibiting naive T cell (Tn) binding to cognate APCs while permitting bystander naive T cell access.^322^

Immune tolerance is a critical process during thymic development and involves thymic selection and TCR recombination.^323^ The first checkpoint used to assess whether developing T cells have successfully rearranged genomic DNA to produce functional TCR β chains is termed the β-selection checkpoint.^324^ Although immature ISs lack mature TCR structures and cannot engage in canonical peptide–MHC binding, they were historically considered irrelevant to T cell development.^4^ However, emerging evidence shows that stromal cell-developing T cell interactions at the β-selection checkpoint involve MTOC recruitment^325,326^—a hallmark of IS initiation. Developing T cells employ Notch and CXCR4 signaling to form ISs in vitro and in situ, resembling mature T cells, thereby mediating the pre-TCR complex signaling required for β-selection checkpoint progression.^327^ Single-cell sequencing data revealed that canonical CD8-associated ISs formed between thymocytes at the double-positive cell selection stage,^328^ whereas rapid recruitment of p56(lck) and CD3ζ occurred during the double-negative cell selection stage without full synapse polarization.^15^

These findings illustrate how the immune system employs the IS as a precision tool to orchestrate physiological processes, underscoring its role as a central target for maintaining organismal homeostasis and health.

## Immunological synapses in various diseases

**Fig. 5:**
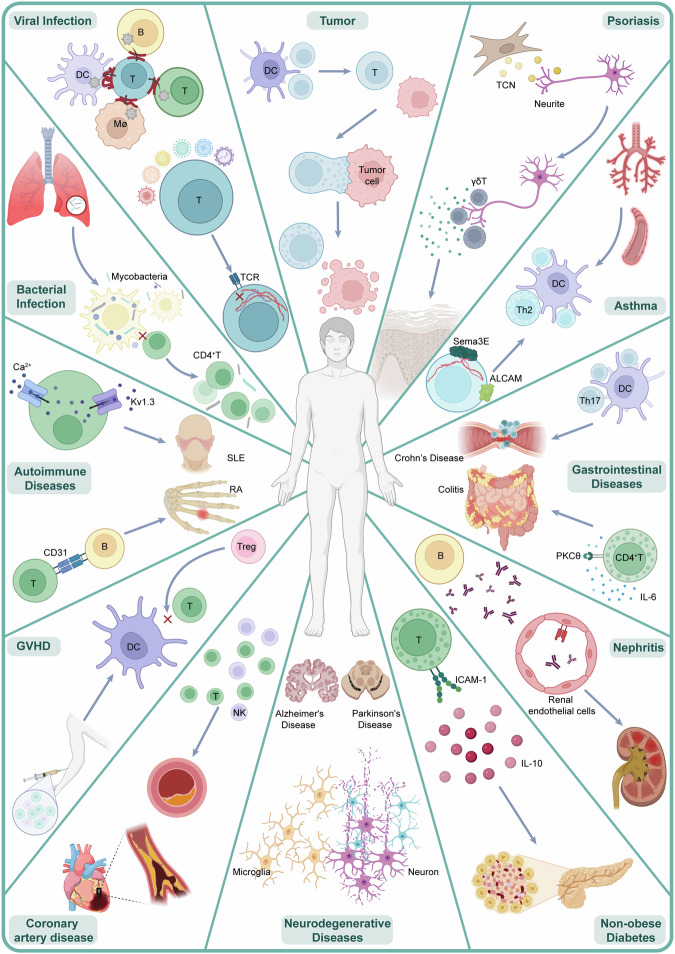
Immunological synapse in various diseases. Dysregulation of immunological synapses is involved in various human diseases. For instance, it is necessary to maintain normal Pre/Post-synaptic T cells to recognize and kill tumor cells. Bacteria and viruses employ various mechanisms to inhibit IS assembly and downstream signal transduction. Viruses can even establish viral synapses to facilitate cell-to-cell infection, thereby evading immune cell-mediated killing and causing infections. However, excessively strong IS can lead to the overactivation of cells, particularly T cells, resulting in excessive cytokine secretion and the development of non-infectious inflammation. For example, an overabundance of IL-17 can trigger conditions such as psoriasis and Crohn’s disease. Overproduction of autoantibodies by B cells can lead to nephritis and rheumatoid arthritis, while overactivation of microglia can result in the phagocytosis of normal neurons, contributing to the development of Alzheimer’s disease and Parkinson’s disease. Additionally, there are therapeutic interventions targeting the formation of IS, such as those used for GVHD and allergic asthma. Image created with BioRender (https://biorender.com/)

The homeostasis of immunological synapses is critical for maintaining organismal health, but their dysregulation underlies the pathogenesis of various diseases (Fig. 5).

### Tumors

**Fig. 6:**
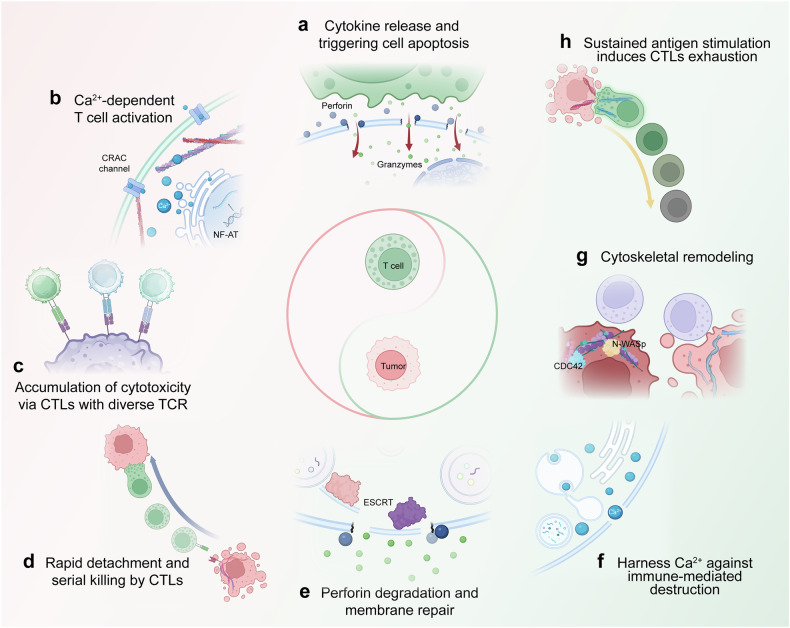
Immunological synapse in modulating tumor immune surveillance and immunoevasion. The yin and yang struggle between T cells and tumor cells at the IS. **a** T cells form an immune synapse with tumor cells, and perforin creates pores in the tumor cell membrane, allowing granzymes to enter the target cell and induce apoptosis. **b** T cell activation depends on the influx of Ca^2+^ and the activation of NF-AT. **c** Diverse TCR-expressing CTLs can bind to the same tumor cell, resulting in a cumulative cytotoxic effect. **d** After attacking, the tumor cell cytoskeleton contracts, prompting the CTL to separate the apoptotic tumor cell, thereby mediating the killing of other tumor cells. **e** After the tumor cell membrane is perforated, ESCRT is recruited to the contact site and mediates membrane repair. **f** Tumor cells utilize Ca^2+^ for membrane repair to prevent immune-mediated destruction. **g** CDC42 and N-WASP remodel the tumor cell cytoskeleton to resist NK cell-mediated killing. **h** Dying tumor cells still form an IS with CTLs, continuously providing antigenic stimulation signals that induce T cell exhaustion. Image created with BioRender (https://biorender.com/)

The neoplasm stands as the paramount focus of research into the IS, a preoccupation owing not only to the considerable portion of the immune-invasive tactics evolved by cancer cells involving the direct or indirect dismantling or weakening of the IS to elude immunosurveillance but also to the burgeoning evidence that the status of the IS is intimately correlated with the prognostic outcomes in patients with cancer. Moreover, targeting the correction of the initiating factors of IS dysregulation has been shown to potently enhance the body’s antitumor immunity, thereby eliminating malignancy. It is reasonable to posit that a certain equilibrium is struck between the body’s tumor immune surveillance and tumor immune evasion, demarcated by the IS—a parity akin to the Tai Chi concept of Yin–Yang balance in classical Chinese Taoism (Fig. 6). We ought to thoroughly comprehend the pivotal mechanisms underpinning this equilibrium and aid in inclining the scales of victory toward anti-tumor immunity.

#### Serial killing of tumors

Despite the heightened sensitivity, swiftness, and effectiveness of LG secretion, a single CTL is inefficient in eradicating tumor cells.^329^ According to bulk cytotoxicity assays and mathematical models, each CTL has the potential to eliminate 1 to 20 cells per day.^329,330^ However, in patients who have received adoptive transfer of tumor-specific TCR-engineered or CAR-T cells, CTL-mediated continuous killing has rarely been observed to be efficient.^331^ In an antigenic solid tumor model with a 1:1 ratio of CTLs to target cells, CTLs predominantly formed transient contact structures with target cells, and very few target cells were directly prompted to undergo apoptosis upon contact.^332^ In vitro live imaging of bone marrow and in vivo single-cell level analysis have validated that the administration of lethal hits is a relatively infrequent result of CTL–tumor cell interactions, whereas the recuperation of tumor cells following sublethal hit delivery is quite prevalent.^79,333,334^

Consequently, disparities in the cytolytic potential of CTLs and the resistance of tumor cells to cytotoxic assaults are critical hurdles in the in vivo anti-tumor response.^334^ The efficacy of lethal hit delivery varies among individual CTLs, as evidenced by the variability in the frequency of lethal/sublethal hits. This variability may stem from differential perforin expression in CTLs, varying intensities of TCR signaling, CTL polarization and degranulation efficiency, and heterogeneity in the uptake of granules by target cells.^335^ Moreover, the repair mechanism in target cells may differ, yielding either lethal outcomes or survival despite comparable frequencies and quantities of perforin-mediated events. The repair of sublethal CTL hits may further potentiate or weaken the susceptibility of target cells to subsequent hits or give rise to mutations, thus manifesting a distinct mode of damage and repair processes.^336^ Notably, it has been postulated that the accumulation of sublethal hits could mediate effective elimination: in a live melanoma tissue model, it was observed that three consecutive hits with decay intervals of less than 50 minutes could effectively induce target cell death.^333^ This underscores the incremental and adjustable nature of CTL-mediated cytotoxicity through multiple hit delivery, emphasizing that increasing the magnitude and frequency of damage can augment immune efficacy.^333^ Additionally, CTLs with diverse TCR specificities may confer increased cytotoxicity, emphasizing the importance of a high local density of antigen-specific CTLs in anti-tumor immunity, as well as the prospects of combined NK/T-cell therapeutic approaches.^337^

Notably, the death of individual tumor cells is not the endpoint of serial tumor killing. The disassembly of the IS, which is mediated by the shedding of CD16, facilitates the detachment of NK cells from target cells, thereby increasing their motility, contributing to their survival, and augmenting their sustained binding to target cells.^130^ After undergoing “altruistic apoptosis”, however, some dying target cells can still become traps that impede CTL killing, aiming to minimize harmful effects on surrounding tissues.^306^ These dying target cells can still form an IS with CTLs and provide continuous signaling when they exhibit sufficiently strong antigen stimulation.^10^ This favors cytokine secretion by CTLs and amplifies CTL responses, ultimately promoting continuous killing by CTLs.^10^ However, this notion has been questioned, as sustained antigen stimulation and cytokine secretion do not necessarily lead to an increase in the overall killing outcome of the same CTL but may instead result in T-cell exhaustion and premature decay.^131^ In contrast, it has recently been demonstrated that contraction of the target cell cytoskeleton occurs after target cell apoptosis, which is a necessary and sufficient condition for the dissociation of CTLs from target cells. Genetic and pharmacological perturbations of target cell cytoskeletal contraction significantly impair the efficiency of rapid detachment and continuous killing by CTLs.^306^ Therefore, CTLs must perceive the exact timepoint at which their job of delivering the final blow to target cells has been completed, which is undoubtedly meaningful for increasing the overall efficiency of tumor clearance. However, the factors that determine this perception mechanism and its true occurrence in vivo are still under investigation.^338^

#### Tumor resistance to immune cytotoxicity

In the context of anti-CD19 CAR-T cell therapy, most interactions between CAR-T cells and tumors are also unrelated to lethal hit delivery.^334^ On one hand, both TCR and CAR-T/NK cells exhibit similar initial kinetics of rapid decay after serial killing, with the latter two even decaying faster. This could be ascribed to the perturbation of synaptic structures following artificially engineered antigen‒antibody-based target recognition.^339^ On the other hand, this sublethal event can be attributed to a spectrum of resistance mechanisms instigated by tumor cells at the IS interface and more extensively within the framework of apoptosis evasion, effectively eliminating CTL attacks.^21^ The characterization of sublethal damage types includes reversible perforin-mediated pore formation, nuclear membrane rupture, and DNA double-strand breaks, with target cells being able to initiate Ca^2+^-mediated endo-/lysosomal transport-enhanced membrane repair.^340^ The nuclear lamina is subsequently restored by endosomal sorting complexes, which are required for transport-dependent transport, within 30–90 min.^341^ Upon occurrence, DNA double-strand breaks initiate the DNA damage response (DDR) pathway, leading to cell survival within several hours via non-homologous end joining and homologous recombination-mediated DNA repair.^342^ Additional means employed by tumor cells include perforin degradation-mediated insufficient granzyme B penetration, termination of SNAP-23-dependent lysosomal trafficking, pH disturbance, or impairment of lysosomal proteolytic activity, which can restore susceptibility to CTL attacks.^340^

Among these self-protective mechanisms, membrane softening and membrane repair are pivotal, as membrane damage serves as the initial step in cell destruction. Perforin initiates the killing cascade by forming pores in the target cell membrane, which activates the membrane repair response, leading to the uptake of granzymes into the target cell cytoplasm, where they cleave many key substrates to trigger cell apoptosis.^343^ However, tumor cells can soften their cell membranes by diminishing their cytoskeletal framework^18,19^ or by reducing the cholesterol content within their cell membranes,^227^ thereby diminishing the pore-forming efficacy of perforin. Conversely, targeting F-actin to increase cytoskeletal rigidity^18,344,345^ or employing ACAT1 inhibitors to increase membrane cholesterol levels and harden cells^226,227^ can markedly augment the cytotoxicity of immune cells to cancer cells. Tumors also possess a mechanism for self-repair of their cell membranes, and excessive membrane repair may result in the failure of perforin pore formation, aiding these target cells in resisting CTL attacks. The highly increased lysosomal content is closely associated with membrane repair, as evidenced by the gradual increase in the intracellular expression of the lysosomal marker CD107a from the center to the periphery of melanoma tumor nodules in Sox10-high melanoma cells.^95^ Membrane repair also requires the involvement of ESCRT proteins.^346^ After perforin enters the target cell, ESCRT proteins are precisely recruited to the CTL contact site, where they participate in the repair of perforin pores and limit the entry of granzymes into the target cell. Thus, inhibiting the ESCRT mechanism in cancer cells is believed to increase their susceptibility to CTL-mediated killing.^347^

Furthermore, the competition between CTLs and their target cells for Ca^2+^ also represents a pivotal juncture in dictating the trajectory of the immunological battle toward either immune clearance or immune evasion. The functional integrity of the Ca^2+^ pathway is indispensable for mediating the release of granules, with the activity of the CRAC channel governing the individual’s contact potency and the cooperative action of CTLs crucial for the sustained elimination of target cells.^348^ This is underscored by the diminished CTL-mediated lethal impact observed in patients with deficient Orai-STIM Ca^2+^ channels.^349^ In the context of tumor cells, perforin-dependent Ca^2+^ influx during the early stages of target cell entry synergistically triggers apoptosis.^343,350^ However, subsequent exposure to lysosomes and subsequent endosomal-based membrane repair endow tumor cells with the ability to harness Ca^2+^ as a safeguard against immune-mediated destruction.^95,351^

#### Tumor microenvironment

The tumor microenvironment (TME) assumes a pivotal role as a conduit for diverse information exchanges among distinct entities.^352^ Direct interchanges among T and NK immune cells and their target counterparts, encompassing antigen-presenting cells (APCs) and/or tumor cells, engender crucial processes such as antigen presentation, T cell activation, and cytotoxicity, thereby ensuring the seamless progression of anti-tumor immune responses.^353^

Excessive accumulation of certain metabolic substances within the TME may impair the transport and assembly of essential components required for the formation of the IS. Dysregulation of serine metabolism in tumors leads to a reduction in the levels of sphingomyelin in NK cell membranes, thereby disrupting the membrane topology of cellular protrusions and inhibiting NK cell recognition and the formation of lytic synapses with tumor target cells.^122^ However, lipid overaccumulation in the TME due to obesity also obstructs the degranulation process during synapse formation through PPAR-driven glycolytic inhibition.^354^ N-Acetylaspartate is enriched in the TME of HER2^+^ breast cancer patients resistant to immunotherapy, and it disrupts IS formation by promoting PCAF (acetyl transferase)-induced acetylation of lamin A-K542, inhibiting the integration of lamin A and SUN2 (chromatin reassembly factor), and damaging lysosomal polarization. This leads to a reduction in brain inflammation and impairment of antitumor immunity by inhibiting the cytotoxicity of NK cells and CD8^+^ T cells.^355^ γδ T cells are also victimized by the accumulation of metabolic waste in the TME, as excessive lactic acid significantly reduces the transport of cytotoxic granules to the Vγ9Vδ2 T cell-tumor synapse by inhibiting AMPK activation, resulting in the loss of antitumor activity in vitro, in vivo, and in patients.^356^

The IS also drives changes in immune infiltration patterns within the TME. The membrane tetraspanin claudin (CLDN) 18 drives the translocation of the adhesion protein ALCAM to the lipid rafts of tumor cell membranes (via actin), facilitating direct contact between CTLs and tumor cells.^357^ This process promotes the formation of robust synapses between CTLs and CLDN18-positive cancer cells, leading to increased T cell activation and the transformation of pancreatic ductal adenocarcinoma (PDAC) from an immunological desert to an immune-infiltrated type.^357^ Galactosylceramide-3A (SEMA3A) inhibits the assembly of IS and T cell activation by interacting with neuropilin-1 (NRP1) on the surface of CD8 + T cells, resulting in reduced T cell infiltration into tumors.^345^ The interdependent binding and function of CREBBP and KMT2D on chromatin preferentially impair the activation of super-enhancers responsive to the IS, leading to CD8^+^ T cell exhaustion and reduced infiltration.^358^

Systematic investigations into the differences in IS characteristics among various T cell subsets within the TME are currently lacking. A recent study using super-resolution microscopy and computational simulation analysis in the classic lymphocytic choriomeningitis virus (LCMV) mouse infection model revealed significant differences in key parameters of IS formation (compressive strength and protrusive activity) among different T cell subsets: Tn < Tex ≈ Memory T cell (Tm) < Teff. Although Tm and Tex exert intermediate levels of compression, Tex cells generate fewer protrusions than even Tn cells do.^62^ Additionally, α-SMA^+^ CAFs can form ISs with Foxp3^+^ Tregs to guide the immobilization, activation, and proliferation of Tregs, thereby promoting immune tolerance to tumors.^359^

#### Tumor metastasis

Recent studies have underscored the importance of target cell rigidity as a pivotal factor controlling the magnitude of synaptic force, as well as its impact on the formation and activity of lytic synapses.^19^ Target cells can evade the killing of CD8^+^ T cells through mechanics-driven softening mechanisms controlled by cytoskeletal proteins, which act as barriers to the formation of perforin pores at the synapse site.^19^ The implications of target cell rigidity are particularly relevant to tumor metastasis, as cancer cells tend to soften themselves to withstand various hemodynamic and interstitial fluid pressures during the metastatic cascade and acquire invasiveness, primarily through alterations in their cytoskeletal structure.^360–362^

Notably, this process is considered a key driver of tumor immune evasion,^363^ and conversely, enhancing the stiffness of cancer cells can reduce tumor metastasis by augmenting immune cytotoxicity,^344^ possibly by redirecting the actin cytoskeleton of cancer cells to the IS.^17^ In alignment with this, melanoma cells that survive CTL cytotoxic attacks exhibit distinct transcriptomic features pertaining to focal adhesion, trans-endothelial migration, and cytoskeletal remodeling, leading to enhanced migratory and invasive properties both in vitro and in vivo.^364^ Even for tumor cells that have already metastasized, IS-mediated immune surveillance remains crucial for controlling tumor metastasis.^20^ If the assembly of synapses in peripheral blood, which are cytotoxic immune cells, can be effectively stimulated, it can significantly clear circulating metastatic tumor cells.^20,121^

#### Tumor prognosis

We previously mentioned numerous factors that can lead to dysregulation of the IS, whose expression in tumor cells is often significantly correlated with prognosis, including ICAM1,^365^ MRTF,^344^ SEMA3A,^345^ NAT8L,^355^ CLDN18,^357^ MAFG^362^ and so on. Recently, a subset of circulating tumor cells (CTCs) encapsulated by EV-derived CD45 (referred to as CD45^+^ CTCs) was identified. They prevent the exclusion of CD45 from the TCR-pMHC synapse through intercellular CD45-CD45 homophilic interactions with CD45^+^ T cells, leading to weakened TCR signaling and suppression of the immune response.^20^ Consequently, the detection of CD45^+^ CTCs in the bloodstream can also be used to assess patient prognosis.^20^

Advancements in imaging technologies, spatial transcriptomics, and computational analysis have recently made it possible to directly evaluate the formation of the IS. Super-resolution traction force microscopy can significantly differentiate synapses among various cell types; for example, T cell synapses are generally contractile, which is distinctly different from the pulling and gripping associated with macrophage phagocytic synapses.^62^ Repeated cell neighborhoods involving tumor cells, immune cells, and stromal cells undergo significant changes along the progression axis involving precursor states, in situ melanoma, and invasive tumors, with stronger T cell and tumor cell synapse interactions typically observed in areas of tumor regression.^366^ Recently, researchers have presented a novel approach for multipronged computational immunological synapse analysis (CISA) that reveals T-cell synaptic interactions from multiplex images.^61^ CISAs are capable of automatically detecting and quantifying synapse interactions on the basis of the localization of proteins on the cell membrane.^61^ Moreover, CISA is universal and can be extended to immunocyte clusters in various tumors, such as breast cancer and melanoma, and CISA quantification of T cell:B cell synapses can predict improved patient survival rates.^20^

### Infectious diseases

#### Bacterial infection

To establish disease or chronic infections, pathogens must overcome both the innate and adaptive immune defenses of the host simultaneously. The IS represents a critical target for pathogens to manipulate host immune responses. Evasion of innate immunity often involves exploiting innate immune cells such as macrophages, which are inhibited by specific virulence factors from killing internalized bacteria, thereby creating a protected niche for pathogen replication.^300^ For *example, mycobacteria* secrete mannosylated lipoarabinomannan (ManLAM), which prevents infected macrophages from forming functional ISs with CD4^+^ T cells and suppresses T cell activation and IL-2 secretion, facilitating immune evasion of *M. tuberculosis*.^367^ Other pathogens employ mechanical forces to block receptor activation by preventing spatial segregation of phosphatases at phagocytic synapses, leading to pathogen internalization into vacuoles that fail to mature into degradative compartments, thereby evading lysosomal destruction.^368^

IS assembly is a pivotal event in T cell-mediated adaptive immunity. Consequently, many pathogens have evolved mechanisms to disrupt this process, either by indirectly impairing the ability of APCs to present antigens to T cells^369–372^ or, as emerging evidence suggests, by directly inhibiting synapse assembly in T cells.^373–377^ A shared strategy among pathogens involves interfering with MHC class II (MHCII) loading and trafficking to suppress antigen presentation.^369,370^ For example, *Helicobacter pylori* disrupts the proteolytic generation of T cell epitopes for loading onto nascent MHCII molecules, specifically inhibiting the invariant chain (Ii)-dependent pathway via its major virulence factor, vacuolating cytotoxin A (VacA).^378^

#### Viral infection

Numerous viruses have evolved shared strategies to directly inhibit IS assembly, such as suppressing TCR downstream signaling^375^ or blocking actin remodeling.^376^ Examples include *herpesviruses*,^379^ *measles virus*,^380^ *herpesvirus saimiri* (HVS),^381^ *human immunodeficiency virus* (HIV),^382^ *respiratory syncytial virus* (RSV),^383,384^ *heatitis C virus*,^385^ *Epstein–Barr virus* (EBV),^386^ and *SARS-CoV-2*.^387^ Bacterial pathogens employ analogous tactics, such as targeting TCR and co-stimulatory signaling pathways, as exemplified by *Neisseria gonorrheae*,^388^ *Fusobacterium nucleatum*,^374^ *Neisseria meningitidis*, *Haemophilus influenzae*, *Moraxella catarrhalis* (by disabling CEACAM1),^389^ *Mycobacterium tuberculosis* (degrading CD3β),^373^ *M. tuberculosis* (inhibiting LCK translocation to synapses),^367^ and *Helicobacter pylori* (blocking the Ca²⁺-calcineurin pathway).^390^ To disrupt cytoskeletal dynamics, *Shigella* promotes T cell cytoskeletal accumulation via its T3SS effector, impairing IS formation during APC scanning,^377^ whereas *M. tuberculosis* activates cofilin through MtSerB2 to perturb F-actin remodeling.^391^ For centrosome polarization, *Salmonella* forces expression of the protease GtgE to cleave and inactivate Rab29 and Rab8, suppressing the vesicular trafficking required for synapse assembly.^392^ Notably, bacterial strategies often align with survival needs: *Staphylococcus aureus* sepsis-associated superantigens (SEA, SEB, TSST-1) drive antigen-independent TCR binding, inducing hyperstable synapses that trigger T cell overactivation and cytokine storms.^393^

Beyond disruption, the IS can be hijacked into “virological synapses” that facilitate viral spread.^394^ In vitro, in vivo and clinical evidence confirms that HIV-1, HTLV-1, and encephalitis viruses repurpose synapses into shielded hubs for intercellular viral transfer, evading antibody neutralization and complement recognition.^395–399^ The transmission routes include DC-to-T cells,^371,372^ T-to-T cells,^400^ monocyte/macrophage-to-T cells,^164^ and B-to-T cells.^401^ Viral transfer from APCs to T cells occurs via two modes: cis-infection (viral replication in immature DCs followed by progeny release)^402^ and trans-infection (mature DCs transport intact virions to T cells without replication).^403,404^ The key steps in virological synapse formation include gp120-receptor binding,^405^ DC-SIGN-mediated virion adhesion,^403^ and membrane protrusion/contact.^406^ These mechanisms reflect viral cooption of the synapse machinery: while TCR signaling is suppressed to reduce antiviral affinity and limit T cell activation,^407^ viruses exploit the inefficient activation of T cells to increase infection and replication efficiency.

### Non-infectious inflammation

Although endothelial cells are not professional APCs, most endothelial populations, including renal and vascular endothelial cells, constitutively express MHCII antigens under cytokine stimulation.^408^ Recent studies have revealed that augmented mechanical stretch in endothelial cells contributes to the pathogenesis of numerous cardiovascular and renal disorders.^409^ In this context, increased mechanical stimulation of endothelial cells has been shown to promote IS formation, exacerbating renal inflammatory diseases.^409^

Chronic skin inflammation is the main clinical symptom of psoriasis, and overactivation of the immune system is an important cause of its onset; however, its exact origin is still unclear. Single-cell omics profiling of fibroblast lineages revealed a Tnc^+^ papillary fibroblast subset with pro-axonal and neuromodulatory transcriptional signatures. This subset facilitates “neuroimmunological synapse” formation between dermal γδ T cells and aberrant neurites, leading to excessive production of type 17 pathogenic cytokines.^24^

The IS interaction between airway DCs and T cells is one of the key pathogenic mechanisms of allergic asthma, leading to the excessive activation of T cells and triggering Th2-dependent eosinophilic airway inflammation and bronchial hyperresponsiveness.^410^ Targeting actin cytoskeleton remodeling or adhesion molecule‒integrin interactions to reduce the formation of the IS may be an important and feasible approach to improve airway inflammation in patients with asthma.^411^

### Neurodegenerative, neurodevelopmental and cerebrovascular diseases

The intricate interplay between the nervous and immune systems is exemplified by microglia, the resident innate immune cells of the brain. Dysregulated synaptic phagocytosis or hyperactivation of microglia is recognized as a critical pathogenic mechanism in numerous neurological diseases. As a leading cause of adult disability, ischemic stroke induces progressive neuronal death and synaptic loss. Microglial phagocytosis of stressed synapses exacerbates the disruption of surviving neuronal networks and associated brain functions.^412^ This detrimental activity may arise from disease-induced inflammation, subtoxic injury, or the release of chemoattractants from neighboring phagocytosed dead cells, which impair the capacity of microglia to distinguish viable versus dying neurons.^413^ Endogenous microglia respond to neuronal damage by phagocytosing synapses in reactive gliosis zones, thereby hindering brain repair. Targeted inhibition of phagocytic reactive microglial proliferation reduces synaptic loss and improves post-stroke recovery.^414^

Alzheimer’s disease (AD) is characterized by the pathological accumulation of plaques containing Aβ, phosphorylated tau (p-Tau), and synaptic debris.^415^ Microglia play a dual role in AD: promoting Aβ clearance during the early stages, whereas complement system activation later triggers excessive synaptic phagocytosis that engulfs viable neuronal synapses, thereby contributing to cognitive decline.^416^ As the second most common neurodegenerative disorder, Parkinson’s disease (PD) involves degeneration of dopaminergic neurons in the substantia nigra, leading to depletion of striatal dopamine (DA).^417^ Microglial hyperphagocytosis of dopaminergic neurons represents a key pathogenic mechanism in which α-synuclein (α-Syn)-induced microglial activation via Fcγ receptors exacerbates DA neuron loss and neurodegenerative progression.^25^

Multiple sclerosis (MS) is a neuroinflammatory disorder of the central nervous system (CNS), with over 50% of patients experiencing cognitive impairment.^418^ A study analyzing postmortem hippocampal samples from 16 MS patients revealed that cognitive deficits in MS correlate with excessive microglial phagocytosis of C1q-tagged inhibitory synapses. Additionally, these microglia were found to exhibit hyperactivated direct contact with CD8^+^ T cells, although definitive evidence of T cell-microglial IS remains unproven.^419^ Parallel research revealed that during disease relapse, Th17 cells form synaptic-like structures with neurons, which are associated with extensive axonal damage. Autism spectrum disorder (ASD), a neurodevelopmental condition, involves inactivating mutations in the microglial receptors TREM2 and TMEM59. These defects impair the phagocytic clearance of toxic aggregates or apoptotic membranes, disrupting synaptic pruning and neural circuit remodeling during critical early stages of brain development.^420,421^

### Autoimmune disorders

The hyperactivation of autoreactive B cells is a central pathogenic mechanism in autoimmune disorders such as membranous nephropathy^422^ and systemic lupus erythematosus (SLE).^423^ In these conditions, increased phosphorylation of the IgG1 immunoreceptor tyrosine-based tail (ITT) motif in B cells markedly prolongs the dwell time of the adapter protein Grb2 at the IS, driving excessive B cell activation and triggering broad-spectrum autoantibody production.^423^ In SLE, aberrant activation of T cell calcium signaling and Kv1.3 channel trafficking to synapses further promotes T cell hyperactivity and immune inflammation.^23^

Analysis of synovial biopsies from patients with autoimmune or rheumatoid arthritis (RA) revealed dysfunctional co-inhibitory receptor CD31 on T cells. Strikingly, the administration of CD31 agonists blocks the formation of functional T/B immunological synapses, attenuating pathogenic T-B cell interactions and alleviating clinical arthritis symptoms.^424^ The pathogenesis of demyelinating autoimmune diseases involves hyperactivated neutrophils that transiently acquire immune-stimulatory and MHCII-mediated antigen-presenting capacities, termed ICAM1^+^ macrophage-like neutrophils. These cells form excessive ISs with T and B cells, perpetuating chronic inflammation.^425^

### Metabolic dysregulation and immunodeficiency disorders

IL-10 secretion in pancreatic islets plays a critical role in the pathogenesis of nonobese diabetes.^426^ One proposed mechanism involves the upregulation of ICAM-1 expression, which promotes IS formation, driving T cell hyperactivation and excessive inflammatory cytokine release that damages islet cells and the vascular endothelium.^48^ Phenylketonuria (PKU) is a metabolic disorder caused by dysfunctional phenylalanine hydroxylase (Pah), leading to elevated phenylalanine levels in the brain and intellectual disability.^427^ In the *Pah*^enu2^ mouse model (carrying a mutated Pah gene), phenylalanine feeding reduces microglial synaptic pruning capacity,^428^ potentially resulting in excessive neuronal density and impaired cognitive development.^429^

Wiskott–Aldrich syndrome (WAS) is among the earliest studied disorders linked to IS dysregulation.^430^ T cells deficient in WASP form conjugates with APCs at normal frequencies but extend actin-rich protrusions away from synapse regions. Their MTOCs fail to polarize toward synaptic centers, disrupting synapse architecture and signal transduction and ultimately impairing T cell activation and cytokine production.^431^ WHIM (warts, hypogammaglobulinemia, infections, myelokathexis) syndrome is a rare disease characterized by diverse symptoms indicative of aberrantly functioning immunity. Patients with mutations in the chemokine receptor CXCR4, which prevents normal recruitment to the IS, exhibit destabilization of T-APC conjugates, leading to delayed IgG class switching and impaired memory B cell function.^186,432^

### Gastrointestinal and cardiovascular disease

Crohn’s disease, a chronic granulomatous inflammatory disorder affecting the entire gastrointestinal tract, arises from loss of immune tolerance and aberrant activation of mucosal immunity.^433^ DCs typically generate synapse-directed autophagosomes formation to negatively regulate T cell activation. However, ATG16L1 and IRGM gene variants in Crohn’s disease patients impair autophagy, preventing elimination of DC-T cell synapses and resulting in Th17 hyperactivation and tolerance breakdown.^434^

Protein kinase C (PKC)θ, a critical mediator of IS formation and T cell activation, is indispensable for colitis pathogenesis. PKCθ-deficient mice fail to develop Th1/Th2-mediated colitis, underscoring its central role.^435^ The epithelial lectin galectin-4 induces synapse formation via PKCθ-dependent pathways, specifically stimulating IL-6 production in CD4^+^ T cells. Under inflammatory conditions, galectin-4-mediated CD4^+^ T cell activation exacerbates chronic colitis and delays recovery from acute intestinal injury.^54^ Further studies demonstrated that galectin-4 requires interaction with a specific binding glycome to stabilize active PKCθ, inhibit synapse disassembly, and drive persistent memory CD4^+^ T cell expansion in colitis.^436^

Notably, dysfunctional “neuroimmunological synapses” between the enteric nervous system and immune cells may also contribute to gastrointestinal inflammation and dysmotility.^437^ Unlike classical synapses, this dysregulation involves phenotypic and functional alterations mediated by the secretion of neurotransmitters (norepinephrine, serotonin, acetylcholine) and immune mediators (cytokines, proteases, opioids and neurotransmitters), collectively disrupting local intestinal microenvironments.^438,439^ While neuro-immune-inflammatory crosstalk encompasses vast signaling networks, tracing the spatiotemporal dynamics of these secretory factors remains methodologically challenging, limiting the current understanding of “neuroimmunological synapse”-specific mechanisms.

Coronary artery disease (CAD) is characterized by the accumulation of atherosclerotic plaques in blood vessels that supply oxygen and nutrients to the heart. Its pathogenesis is classically attributed to endothelial dysfunction of coronary arteries and lipoprotein infiltration into the vessel wall.^440^ Recent whole-blood RNA sequencing in CAD patients revealed significant T cell dysregulation in moderate-to-severe cases, with upregulated transcription of genes associated with ciliary function and IS formation, suggesting novel mechanisms underlying this prevalent cardiovascular disorder.^441^ Intriguingly, similar transcriptional alterations were observed in Treg cells.^442^ Elevated inflammatory markers linked to autoimmunity have also been detected in CAD patients,^443^ and multi-omics analyses further confirmed associations between T/NK cell-mediated inflammation and adverse clinical outcomes.^443^ These findings collectively implicate IS hyperactivation as a potential driver of CAD pathogenesis and disease progression.

## Therapeutic interventions

### Targeting immunological synapse formation

As previously mentioned, the key mechanisms regulating IS formation and disassembly include cytoskeletal remodeling, membrane reshaping, force signal transduction, and integrin/adhesion signal transmission. Therefore, we subsequently discuss the therapeutic strategies targeting IS formation from these four aspects (Table 1).

**Table 1:** Summary of pre-clinical therapeutic targets for the treatment of immunological synapses

Treatment varieties	Specific targets/drugs	Species	Target/Disease of interest	Year	Reference	Function
Pre-clinical studies targeting IS formation						
Targeted drugs	Lenalidomide	Human	Multiple myeloma	2015	Lagrue et al.^121^	Increase the periodicity of cortical actin; Promote IS formation
	Laponite	Mouse	Hepatitis B infection	2023	Li et al.^445^	Induce cytoskeletal reorganization of DCs; Increase DC-T-cell synapse formation
	MRT-6160 (Targeting VAV1)	Mouse	Arthritis/Colitis	2023/ 2024	Cartwright et al.^447,448^	Reduce cytoskeletal polymerization; Inhibit IS formation
	Avasimibe (ACAT1 inhibitor)	Mouse	Melanoma/Hepatocellular carcinoma/Glioblastoma	2016/ 2019/ 2020	Yang et al.; Jiang et al.; Hao et al. ^226,449,450^	Increase cholesterol level of T cell plasma membrane; Promote IS formation
	7-ketocholesterol	Human	Breast cancer	2021	Li et al.^451^	Disrupt membrane packing of tumors; Sensitize tumors to NK cell kiling
	IL-15/2-PEG-PTMC	Mouse	Breast cancer	2023	Shao et al.^452^	Promote the formation of lipid rafts; Activate IS formation
	LCL 521 and GW 4869	Human	Hepatocellular carcinoma	2023	Zheng et al.^122^	Inhibit the degradation of NK membrane sphingolipids; Promote IS formation
	2-deoxy-d-glucose (2DG)	Mouse	Lung/ovary/bladder cancer	2022	Greco et al.^453^	Disrupt extracellular N-glycan cover of tumor cells; Promote IS formation
	Epratuzumab	Human	Systemic lupus erythematosus	2013	Rossi et al.^454^	Reduce CD19, CD21, and CD79b via trogocytosis during synapse formation
	T11TS/S-LFA-3/S-CD58	Rat	Glioma	2014	Chaudhuri et al.^455^	Repair key components of the IS at the T-cell-APC interface
	Catch bond engineering	Human	Melanoma	2022	Zhao et al.^456^	Tune high-sensitivity TCRs during synapse formation
	PLP-CD11a BPI	Mouse	Experimental autoimmune encephalomyelitis	2007	Kobayashi et al.^462^	Target integrins and inhibit IS formation
	Efalizumab	Human	Psoriasis	2010	Koszik et al.^465^	Block migration and interfere with the IS
Pre-clinical studies targeting IS structure						
BiTEs/TriTEs/TetraTEs	(4D5/CD3ε or CD19/CD3ε or PSMA/CD3ε or EGFR/CD3ε) BiTE-Sialidase	Mouse	Breast cancer/leukemia/prostate cancer/melanoma	2024	Yang et al.^27^	Bridge tumor and T cells; Promote binding; Enhance T cells’ cytotoxicity
	PD-L1/CD3ε BiTE	Human	Colorectal carcinoma	2021	Khalique et al.^29^	
	PD-L1/CD33 BiTE	Human	Acute myeloid leukemia	2023	Marcinek et al.^539^	
	PD-L1/HLA-G/CD3ε TriTE	Mouse	Heterogeneous lung cancer	2024	Lin et al.^472^	
BiKEs/TriKEs/TetraKEs	CD133/CD16 BiKE	Human	Colorectal carcinoma/Burkitt’s lymphoma	2024	Schmohl et al.^478^	Bridge tumor and NK cells; Promote binding; Enhance NK cells’ proliferation and cytotoxicity
	CLEC12A/IL15/CD16 TriKE	Mouse	Acute myeloid leukemia	2021	Arvindam et al.^479^	
	HER2/IL15/CD16 TriKE	Human	Ovarian cancer	2021	Vallera et al.^480^	
	PSMA/IL15/CD16 TriKE	Mouse	Prostate cancer	2024	Phung et al.^482^	
	CD33/IL15/CD16 TriKE	Human	Acute myeloid leukemia	2016	Vallera et al.^481^	
	EPCAM/IL15/CD16 TriKE	Human	Colorectal carcinoma	2016	Schmohl et al.^483^	

#### Targeting cytoskeletal remodeling

There are numerous inhibitors/agonists that target the cytoskeleton, but owing to their broad target specificity and potential for high toxicity, most of them are not suitable for clinical treatment. Therefore, exploring the additional effects of existing clinical drugs on the cytoskeleton is currently the mainstream approach. Lenalidomide, an immunomodulatory drug widely used to treat multiple myeloma (MM), can significantly increase the periodicity of actin in the NK and T cell cortex, leading to an increase in the area of the actin network and enhanced degranulation of cytotoxic cells, suggesting potential new applications in other tumors.^121,444^ Additionally, targeted nanomaterial-based small-molecule drugs can be developed to avoid off-target toxicity. For example, laponite can induce cytoskeletal reorganization in DCs through the lysosomal reprogramming-calcium flicker axis, increasing the formation of DC-T cell synapses and promoting the activation of antigen-specific CD8^+^ T cells.^445^

VAV1 can mediate the cytoskeletal remodeling of various immune cells and is an indispensable molecule for the formation of IS structures.^446^ Given that the expression of VAV1 is highly restricted to hematopoietic cells, interventions targeting VAV1 can directly influence the pro-inflammatory response while avoiding collateral damage to non-immune cells.^26^ Targeting VAV1 can impede the reactivation of antigen-mediated autoreactive T cells and suppress the secretion of pro-inflammatory cytokines that drive inflammation, tissue injury, and disease progression.^26^ Therefore, a molecular glue degrader, MRT-6160, that targets VAV1 has been developed, and it has been reported that it can inhibit T cell proliferation and cytokine production in a dose-dependent manner.^447^ Orally administered MRT-6160 has demonstrated promising pre-clinical activity in mouse models of T and T/B cell mediated diseases, as it inhibits the progression of collagen-induced arthritis^447^ and colitis.^448^

#### Targeting membrane reshaping

Avasimibe, an ACAT1 inhibitor, can increase cholesterol levels on the surface of CD8^+^ T cells, leading to enhanced TCR clustering and signaling, as well as more effective formation of the IS, thereby augmenting antitumor immunity and inhibiting tumor growth.^226,449,450^ Similarly, NK cells are protected by dense and highly ordered synaptic lipid membranes, and increasing the cholesterol content on the surface of NK cells also aids in their ability to kill tumors.^451^ The use of IL-15/2-PEG-PTMC small molecule polymers can significantly promote cholesterol accumulation and lipid raft formation in NK cell membranes, thereby effectively activating the IS and enhancing NK cell anticancer activity.^452^ In addition to cholesterol, sphingolipids are crucial for NK cell membranes. LCL521 and GW4869 are acidic and neutral sphingomyelinase inhibitors, respectively, which can significantly inhibit the degradation of sphingolipids on the surface of NK cell membranes, thereby increasing the number of membrane protrusions, indicating increased NK cell proliferation and antitumor toxicity.^122^

Carbohydrates on the cell surface can also affect the formation of the IS. For example, the glucose/mannose analog 2-deoxy-D-glucose (2DG) is a mannose analog that can competitively inhibit the generation of extracellular N-glycans on tumor cell membranes, which are overexpressed in various tumors.^453^ Interfering with the accumulation of this metabolite at the IS can significantly promote synapse formation, increase T-cell cytokine production and cytotoxicity, and enhance transcriptional activation.^453^ The humanized anti-CD22 antibody epratuzumab mediates Fc/FcR-dependent trogocytosis from B cells to effector cells, leading to a remarkable reduction in the surface levels of multiple BCR regulatory factors (including CD22, CD19, CD21, and CD79b) as well as crucial cell adhesion molecules (including CD44, CD62L, and β7 integrin) on peripheral blood mononuclear cells derived from normal donors or patients with SLE.^454^ Since the TCR is considered to downregulate signals through membrane shedding after multiple activations,^218,233^ leading to the inhibition of IS formation, a study developed a novel glycopeptide, T11TS/S-LFA-3/S-CD58, which offers a multi-targeted approach for the repair of some key components of the IS at the T cell-APC interface, thereby enhancing the anti-glioma response.^455^

#### Targeting force signaling

Reports on the use of mechanical signals to regulate the IS for immunotherapy are relatively rare and can be divided into two categories. One approach indirectly influences mechanics during synapse formation by regulating the cytoskeleton to control the stiffness of immune cells and target cells.^446^ The other involves directly modifying the catch bonds of the TCR and BCR (since they are considered mechanical sensing receptors), thereby modulating immune responses by inhibiting or promoting the formation of the IS.^456^ Engineered TCR and chimeric antigen receptor T (CAR-T) cell therapies are the ones most likely to benefit from this strategy,^457^ as an important research direction in the past decade has been to increase therapeutic efficacy by increasing TCR/CAR affinity.^458^ However, merely increasing the affinity of TCRs or CAR structures not only fails to proportionally increase the efficacy of immune cell therapies^459^ but also geometrically increases the probability of side effects owing to an elevated risk of cross-reactivity with off-target antigens.^460^ Some studies have isolated TCR mutants that display high activation signals along with low-affinity pMHC binding, successfully enhancing target cell killing activity and reducing undetectable cross-reactivity.^456^ Therefore, future engineering strategies should strike a balance between the threshold of antigen recognition and the kinetics of intercellular conjugation to meticulously calibrate next-generation CAR designs aimed at achieving ideal treatment effects and optimizing the management of off-target toxicity.

#### Targeting integrin and adhesion signaling

The bifunctional peptide inhibitor (BPI) employs ICAM-1-binding peptides to target antigenic peptides (e.g., proteolipid peptide, glutamic acid decarboxylase, and type II collagen) to APCs, thereby modulating the immune response.^461–463^ The central hypothesis posits that BPI impedes the formation of the IS by concurrently binding to MHCII and ICAM-1 on APCs and selectively altering the activation of T cells from the Th1 phenotype to the Treg and/or Th2 phenotype, leading to tolerance.^461–463^ A primary cause of autoimmune diseases is excessive activation mediated by the IS.^464^ Various disease-modifying therapies contribute to the regulation of the IS, facilitating the management of these ailments. Efalizumab, a monoclonal antibody targeting CD11a, which, together with CD18, constitutes a crucial part of lymphocyte LFA-1, inhibits the assembly of the IS by modulating the activation of T cells through integrin signaling,^465^ has achieved success in clinical trials for treating patients with moderate to severe psoriasis.^466^

### Targeting immunological synapse structures

The formation of the IS involves the binding of various ligands and receptors. Conversely, by employing artificial means to connect ligand–receptor signals (bispecific engagers) or modify the structures of ligands and receptors (CAR structures), promoting or inhibiting their binding can also influence the formation of the IS. Bispecific T-cell engagers (BiTEs) are prototypical and structurally simplest bispecific antibodies devoid of the Fc region, comprising two single-chain variable fragments (scFvs) linked by short peptide chains, retaining only the two antigen-binding sites necessary for function. TriTEs and TetraTEs are targeted against three or more components.^27–30^ They can promote cancer treatment by bridging tumor cells and T cells, targeting cancer cells with nanobodies that bind to T cells, or indirectly blocking inhibitory ligand‒receptor interactions. This therapeutic antibody has garnered increasing favor in recent years because of its ability to optimally form an IS between target cells and T cells, thereby unleashing the maximal cytotoxic potential of T cells.^27^ CAR immunotherapy harnesses the power of genetically engineered immune cells expressing unique cell surface receptors, combining tumor antigen specificity with immune cell activation. The types and characteristics of the synapses formed between CAR lymphocytes and target tumor cells are of paramount importance to the anti-tumor efficacy of CAR lymphocytes.^467^

#### Nanobody-based T cell activators: BiTEs, TriTEs, and TetraTEs

BiTEs targeting T cell co-stimulatory molecules such as CD3, CD28, and 4-1BB are the most prevalent T cell engagers, with these antibodies exhibiting notable promotion of T cell proliferation and activation, and several have been approved for the clinical treatment of hematological diseases.^468–470^ DuoBody-CD40×4-1BB exhibits conditional CD40 and 4-1BB agonist activity, which is strictly dependent on the cross-linking of the two targets. Consequently, DuoBody-CD40×4-1BB enhances DC/T cell IS, induces DC maturation, augments in vitro T cell proliferation and effector function, and enhances the ex vivo expansion of tumor-infiltrating lymphocytes derived from patients.^31^ PD-L1/4-1BB bispecific nanobodies can simultaneously bind to PD-L1 on tumor target cells and 4-1BB on effector cells. Furthermore, in-depth exploration of PD-1/PD-L1 has revealed that part of the inhibition of T cell activity by PD-1 is due to its spatial position within the IS, which suppresses actin remodeling at the synapse and thus hinders the contact with APCs and the release of cytotoxic granules.^471^ Therefore, modifications to traditional PD-1 antibodies can maximize T cell activation and tumor-killing effects by altering the position of PD-1 within the IS and simultaneously inhibiting PD-1/PD-L1 binding.^29,472^

#### Nanobody-based NK cell activators: BiKEs, TriKEs, and TetraKEs

Like T cells, which target the activating site CD16 on NK cells, as well as other tumor and NK cell sites, it is also possible to design Bi/Tri/Tetra-specific NK cell engagers (BiKEs, TriKEs, and TetraKEs) that elicit ISs between tumor cells and NK cells.^473^ BiKEs typically target one end to the CD16 single-chain variable fragment (scFv) on NK cells, while the other end targets specific antigens on tumor cells, such as EpCAM, CEA, EGFR, and HER2.^474–477^ These BiKEs can promote antigen-specific antibody-dependent cellular cytotoxicity (ADCC) but are unable to induce NK cell proliferation. Therefore, researchers have begun to explore TriKEs to increase NK cell activation, proliferation, and survival rates. Generally, the synthesis and assembly of hybrid genes encoding TriKEs are achieved via DNA shuffling and DNA ligation techniques, followed by functional assays to test the specificity, efficacy, proliferative capacity, and cytokine profile of TriKEs. Compared with BiKEs, TriKEs demonstrate greater activity against a variety of different tumors, with degranulation assays showing improved kill rates.^478–480^ Moreover, the multi-target design can incorporate IL15, which promotes NK cell proliferation, endowing them with robust expansion and survival capabilities in vivo.^481,482^ Critically, TriKEs provide a selectively self-sustaining signal at the NK/tumor cell synapse, exhibit pharmacokinetics similar to those of intact IgG antibodies in vivo, and lack off-target effects, resulting in reduced toxicity compared with BiKEs, making TriKEs promising new off-the-shelf cancer treatments.^481,483^

#### Chimeric antigen receptors and immunological synapses

Recently, many studies have proposed that the quality of the IS should be utilized to predict the functionality of CAR therapy before clinical application, including the strength, duration, stability, antigen recognition threshold, and intercellular binding kinetics of the IS formed between immune cells and target cells.^484–486^ Specifically, in vitro assessments of the quality of CAR-mediated synapse formation can be performed by quantifying F-actin, the aggregation of tumor antigens, the polarization of LGs, and the distribution of key signaling molecules within the IS.^486^ Moreover, automated algorithms utilizing machine learning and artificial neural networks can be applied to thousands of CAR IS images to perform boundary/edge segmentation, quantify boundary strength, and calculate boundary strength scores, which have been proven to provide accurate IS quality assessments and have good predictive power for the clinical antitumor activity of CAR therapy.^487^ However, owing to current technological limitations, there are no tools available for real-time detection of CAR IS quality in vivo, which represents a hot research direction for future CAR therapy development.

Notably, the IS formed by CAR molecules differs from the typical TCR synapse, as the synapse in CAR-T cells does not involve the formation of a mature synapse but is composed of convoluted Lck microclusters, which have a lower reliance on LFA-1 and lack adhesive rings.^467,488,489^ Compared with the TCR, this less organized synapse allows for faster CAR-mediated contact and subsequent quicker release of signaling cascades^490^, which is associated with faster secretion of cytotoxic granules and subsequent separation of the CAR from target cells.^491^ Another difference is that the antigen-specific modified scFv structure endows CAR molecules with target-specific cytotoxicity; thus, CAR-mediated cytotoxicity is not MHC restricted.^492,493^ This principle makes the CAR lytic synapse require a higher antigen recognition threshold to initiate killing than the standard IS; hence, second-generation or third-generation CAR-T cells typically incorporate one or two intracellular co-stimulatory (ICS) domains upstream of CD3ζ to provide survival signals and increase efficacy.^494,495^

Numerous co-stimulatory molecules that benefit T cell expansion and toxic activation, such as CD28, 4-1BB, ICOS, and OX-40, have been incorporated into the ICS domain modifications of CAR-T cells.^496^ Different co-stimulatory domain regions are considered to have a significant effect on the affinity of the CAR structure.^497^ Similarly, the use of NK-specific signaling domains (such as 2B4 and DAP10 or DAP12) as ICS domains to customize CAR engineering for NK cells can also increase the antitumor efficacy and interferon-γ secretion of CAR-NK cells.^498,499^

The inefficient killing mediated by CARs may also arise from excessive spatial distance between the target epitope and the CAR within the synapse structure or from inefficient binding of the CAR ectodomain to the target antigen.^339,500,501^ Orthogonal and/or membrane-bound cytokine/cytokine receptor engineering strategies can help address this issue. For example, one can use pharmacological methods (such as azacitidine) to increase the antigen density of CD70 in myeloid tumors while simultaneously designing a set of hinge modification regions to reduce the cleavage of the extracellular portion of CD27.^502^ Modification of the CAR IS can also be achieved by adding a PDZ binding motif (PDZbm) intracellularly, which can facilitate additional scaffold cross-linking, thereby enhancing synapse strength and the quantity and abundance of secreted cytokines, as well as prolonging T and NK cell survival in the solid tumor environment.^484,501^ Other major challenges of CAR therapy include severe off-target toxicity caused by the unavoidable recognition of antigens shared by normal tissues.^503,504^ Despite the utilization of the catch-bond mechanism of synapses we previously described, several logic gates and switch receptor CAR-T cells have been developed.^493,505,506^ The latter is the reversible Boolean logic AND gate intracellular network (LINK) CAR, where CD3ζ is replaced by the intracellular proximal downstream signaling molecules LAT and SLP-76, generating CD19-28TM-LAT and HER2-8TM-SLP-76 CAR-T cells^506^. The LINK CARs induced by CD19^+^ HER2^+^ target cells show improved effector function in vitro and in vivo, as well as a reduction in tumor off-target toxicity.^506^

In addition to CAR-T and CAR-NK cells, researchers in recent years have explored CAR-M therapy, which utilizes the phagocytic synapses of macrophages to engulf specific targets, such as cancer cells and pathogens.^507^ First-generation CAR-M cells, which are based on CD3ζ, enable macrophages to phagocytose tumor cells in an antigen-dependent manner. CAR-Ms can express pro-inflammatory cytokines and chemokines, converting bystander M2 macrophages to M1 macrophages, increasing antigen presentation mechanisms, recruiting and presenting antigens to T cells, and resisting the effects of immunosuppressive cytokines.^507^ Recently, second-generation CAR-M, which involves engineering induced pluripotent stem cell-derived macrophages (iMACs) with CARs containing toll-like receptor 4 (TLR4) intracellular toll/IL-1R (TIR) domains, was proposed.^508^ This tandem CD3β‒TIR dual-signaling CAR design endows iMACs with target phagocytic ability, antigen-dependent M1 polarization and M2 resistance in an NF-κB-dependent manner.^508^

## Clinical trials

To date, there have been a considerable number of clinical trials of therapies that modulate the body’s immune response by regulating the formation and structural assembly of the IS, including FDA-approved drugs for various diseases (Table 2). Blinatumomab, a bispecific T-cell-activating antibody, was reported to significantly increase the overall survival of patients with relapsed, refractory, B-cell lymphoblastic leukemia (measurable residual disease positive^509^ or negative^510^). Tebentafusp, a bispecific (gp100×CD3) ImmTAC, notably improved the condition of HLA-A*02:01-positive adult patients with untreated metastatic uveal melanoma (mUM)^511,512^. Other BiTEs proven effective in clinical trials include AMG 420^470^ (or Teclistamab^513^) for treating multiple myeloma, GEN1046 for treating advanced refractory solid tumors,^514^ AFM13 for treating relapsed or refractory Hodgkin lymphoma,^515^ Tarlatamab for previously treated small cell lung cancer,^516^ and Acapatamab for treating mCRPC.^517^ Notably, AFM13, a CD30/CD16A bispecific stimulator capable of activating both T cells and NK cells simultaneously, did not yield the desired outcomes in relapsed or refractory peripheral T-cell lymphomas.^518^

**Table 2:** Summary of clinical trials targeting immunological synapses

Treatment varieties	Specific drugs/therapies	Disease	Year	Stage	Registration number	Status	Patient outcomes	Reference
BiTEs/ TriTEs/ TetraTEs	CD19/CD3ε BiTE (Blinatumomab)	Relapsed large B-cell lymphoma	2016	Phase II clinical trial	NCT01741792	Completed	Overall Response Rate (ORR) = 43%; Complete Response (CR) = 19%	Viardot et al.^509^
		MRD-negative acute lymphoblastic leukemia	2024	Phase III clinical trial	NCT02003222	Active, not recruiting	The 3-year relapse-free survival (RFS) = 80%	Litzow et al.^510^
	GP100/CD3ε BiTE (Tebentafusp)	Metastatic uveal melanoma	2024	Phase I/II/III clinical trial	NCT02570308; NCT03070392	Completed	Median OS = 17.4 months (95% CI, 13.1–22.8)	Sacco et al.; Hassel et al.^511,512^
	BCMA/CD3ε BiTE (AMG 420 or Teclistamab)	Relapsed multiple myeloma	2020/2021	Phase I clinical trial	NCT02514239; NCT03145181	Completed	AMG420: Response Rate (RR) = 70%; Teclistamab: RR = 65%	Topp et al.; Usmani et al.^470,513^
	DuoBody-PD-L1×4-1BB BiTE (GEN 1046)	Advanced solid tumors (colorectal, ovarian, pancreatic and lung)	2022	Phase I clinical trial	NCT03917381	Completed	Disease control =65.6%	Muik et al.^514^
	DLL3/CD3ε BiTE (Tarlatamab)	Small-cell lung cancer	2023	Phase I clinical trial	NCT03319940	Active, not recruiting	Median OS = 17.5 months (95% CI, 11.4–NR)	Dowlati et al.^516^
	PSMA/CD3ε BiTE (Acapatamab)	Metastatic castration-resistant prostate cancer	2024	Phase I clinical trial	NCT03792841	Completed	Median PSA progression-free survival = 3.3 months (95% CI, 3.0–4.9)	Dorff et al.^517^
BiKEs/ TriKEs/ TetraKEs	CD30/CD16A BiKE (AFM13)	Relapsed or refractory Hodgkin lymphoma	2020	Phase I clinical trial	NCT02665650; NCT01221571	Completed	ORR = 83%;	Bartlett et al.^515^
		Refractory peripheral T-cell lymphoma	2025	Phase II clinical trial	NCT04101331	Completed	ORR = 32.4% (95% CI, 23.7% −42.1%)	Kim et al.^518^
	EGFR/CD16A BiKE (AFM24)	Advanced/ metastatic EGFR-expressing cancers	2021	Phase I/II clinical trial	NCT05099549	Terminated	No results posted	NA
Other bispecific antibodies	CD19/FcγRIIb (Obexelimab)	Systemic lupus erythematosus	2023	Phase II clinical trial	NCT02725515	Completed	Loss of improvement (LOI) time increase (hazard ratio [HR] = 0.53, *p* = 0.025)	Merrill et al.^520^
		IgG4-related disease	2023	Phase II clinical trial	NCT02725476	Completed	RR = 93%	Perugino et al.^521^
	IL4/IL13 (Romilkimab)	Systemic sclerosis	2020	Phase II clinical trial	NCT02921971	Completed	Modified Rodnan skin score (90% CI, −4.32 to −0.31, *p* = 0.0291)	Allanore et al.^522^
Chimeric antigen receptor (CAR) therapies	Anti-CD19 plus dominant-negative PD-1 CAR T	Refractory/relapsed B-cell lymphoma	2021	Phase I clinical trial	ChiCTR1900021295	Unknown status	ORR = 32.4% (n = 7/9)	Liu et al.^523^
	CAR19/IL-15 NK	CD19 + B-cell tumors	2020	Phase I/II clinical trial	NCT03056339	Completed	1-year OS = 68%; PFS = 32%	Marin et al.^524^
	Tandem CD19/CD20 CAR T	refractory/relapsed B-cell lymphoma	2020	Phase I/IIa clinical trial	NCT03097770	Completed	ORR = 79% (95% CI, 60%–92%)	Tong et al.^525^
	B-cell maturation antigen-directed CAR T	Relapsed or refractory multiple myeloma	2021	Phase I/II clinical trial	NCT03548207	Completed	ORR = 97% (95% CI, 91.2–99.4, *n* = 94/97)	Berdeja et al.^526^
	NKG2D CAR T	Multiple myeloma	2023	Phase I clinical trial	NCT03018405	Unknown status	ORR = 25% (*n* = 3/12)	Sallman et al.^527^
	PSAM plus dominant-negative TGF-βRII CAR T	Metastatic castration-resistant prostate cancer	2022	Phase I clinical trial	NCT03089203	Active, not recruiting	Median OS = 15.9 months Median progression-free survival (PFS) = 4.4months	Kloss. et al*.*; Narayan et al.^528,529^
	BCMA-CD19 compound CAR T	Systemic lupus erythematosus	2024	Phase I clinical trial	NCT05474885	Recruiting	No results posted	Wang et al.^530^
	CD19-targeting CAR-T	Severe myositis and systemic sclerosis	2024	Not Applicable	NCT05859997	Enrolling by invitation	Deep remission in all 3 patients	Wang et al.^531^
Antibody targeting integrins	Natalizumab (Standard)	Relapsing-Remitting Multiple Sclerosis	2006	Phase III clinical trial	NCT00027300	Completed	Reduction of progression of disability by 42% (HR = 0.58, *p* < 0.001)	Polman et al.^533^
	Natalizumab (Biosimilar)	Relapsing-Remitting Multiple Sclerosis	2023	Phase III clinical trial	NCT04115488	Completed	Mean difference = 0.17 (95% CI, −0.61 to 0.94)	Hemmer et al.^534^
	Vedolizumab	Crohn’s disease (Induction and Maintenance Therapy)	2013	Phase III clinical trial	NCT00783692	Completed	RR = (39.0% vs. 21.6%, *p* < 0.001) or (36.4% vs. 21.6%, *p* = 0.004)	Sandborn et al.^536^
		Crohn’s disease (Prevent postoperative recurrence)	2025	Investigator-initiated trial (IIT)	EudraCT；2015-000555-24	Completed	Recurrence probability versus placebo = 77.8% (95% CI, 66.4%–86.3%, *p* < 0.0001)	D’Haens et al.^537^
		Ulcerative Colitis	2020	Phase III clinical trial	NCT02611830	Completed	Clinical remission probability reduction = Δ32.3% (95% CI, 19.7%–45.0%, *p* < 0.001)	Sandborn et al.^538^

Bispecific antibodies are not employed solely to enhance the function of the IS. They can also be engineered as antibodies that target and block surface markers on immune cells or antigen precursor cells, thereby impeding the formation of the IS to treat autoimmune diseases.^519–522^ Obexelimab, an investigational bifunctional, non-cytotoxic monoclonal antibody, binds to CD19 and FcγRIIb to suppress B cells, plasmablasts, and plasma cells.^520^ In two phase II clinical trials, obexelimab demonstrated favorable safety, good tolerability, and promising clinical response in the treatment of systemic lupus erythematosus^520^ and IgG4-related disease^521^. Another phase II clinical trial indicated that roilkimab also exhibited beneficial effects on early diffuse cutaneous systemic sclerosis^522^.

CAR therapy, which is based on structural modifications of the IS, is currently most frequently applied in patients with hematological malignancies. Among them, CD19-targeted CAR-T and CAR-NK therapies have been proven to be remarkably effective against B-cell lymphoma^523,524^, including the novel dominant-negative PD-1 armored anti-CD19 CAR T cells^523^ and the allogeneic CD19-specific CAR-NK cells.^524^ However, the high failure and relapse rates after CD19-targeted CAR therapy pose considerable obstacles to overcome. A series of tandem CARs, TanCAR7 T cells with dual-antigen targets of CD19 and CD20, yielded better therapeutic effects in refractory/relapsed B-cell lymphoma via the formation of a superior and stable IS structure.^525^ Patients with multiple myeloma also demonstrated early, profound, and long-lasting responses after B-cell maturation antigen-directed CAR T therapy^526^ and autologous NKG2D-based CAR therapy.^527^ Recent CAR therapies have further achieved remarkable advancements in the treatment of solid tumors; e.g., expression of the dominant-negative factor TGF-βRII in CAR-T cells is feasible and safe for treating metastatic castration-resistant prostate cancer.^528,529^

Patients with autoimmune diseases could also benefit from CAR therapy in two phase I clinical trials, especially those suffering from systemic lupus erythematosus^530^ and severe myositis^531^ caused by autoantibodies produced by B cells and plasma/long-lived plasma cells. Another modality approved by the FDA for treating autoimmune diseases is targeting integrins to inhibit the formation of the IS. Natalizumab, a monoclonal antibody that targets α4-integrin, can effectively inhibit the formation of the IS.^464,532^ In phase III trials, patients with relapsing-remitting multiple sclerosis demonstrated favorable outcomes in safety, tolerability, and immunogenicity assessments after treatment with both standard natalizumab^533^ and the biosimilar natalizumab.^534^ Vedolizumab, a monoclonal antibody targeting β7 integrin^535^, promoted remission^536^ and prevented endoscopic recurrence^537^ in patients with Crohn’s disease in a double-blind, randomized, placebo-controlled trial. In another phase III double-blind, double-dummy trial, patients with ulcerative colitis also exhibited favorable safety and tolerability profiles in the treatment group with vedolizumab^538^.

## Conclusion and perspectives

The term “immunological synapse” was initially modeled after “neuronal synapse,” given the analogous framework and membrane structural alterations, the direct exchange of information between two cells, and the secretion of proteins from the pre-synaptic cell to the postsynaptic cell. Consequently, many molecules that regulate the structure of neuronal synapses play pivotal roles in the assembly and disassembly of the IS. However, IS lacks the electrophysiological properties that neuronal synapses possess—or at least none have been discovered thus far—nor do they have the complete structure of axons, dendrites, somata, or the unidirectional transmission characteristics of neuronal synapses. Indeed, the IS often facilitates bidirectional communication through direct contact or secretory particles to regulate organic immune homeostasis and surveillance. This distinctive feature is especially crucial for investigating the role of the IS in pathological conditions, as we must always be mindful and vigilant: tumor and pathogen targets are not inert, passive entities, but rather are critical.

Recent studies have identified the dysregulation of the IS as a significant strategy for immune evasion.^122,227,289,306,351,355,357^ This includes methods such as reducing the cytoskeleton and increasing membrane fluidity to soften cells, diminishing cellular biomechanical stress, enhancing membrane repair, reducing the expression of integrin ligands, and competing for calcium ions. Thus, the formation or dysregulation of the IS is indicative of the success or failure of the struggle between immune cells and tumor cells, a result that may even follow the classical events that mediate immune evasion, such as T cell exhaustion, the formation of inhibitory metabolic microenvironments, and negative regulation by immunosuppressive cells. Hence, it is judicious to posit that forthcoming investigations in the realm of tumor immunology will progressively apprehend IS as an innovative and consequential phenotypic indicator. Moreover, a therapeutic paradigm focused on promoting/inhibiting the formation of the IS and remodeling IS structures may yield profound insights for the advancement of immunotherapy.

The unknowns surrounding the IS are still myriad, a crucial factor being the nascent nature of the research methodologies and technologies, especially since the field of immunity intersects with disciplines such as biophysics and bioimaging. With the advancement of research methodologies, several pending key issues may be resolved: What are the spatial changes in ligands during the formation and dissociation of the IS? What is the relationship between the IS force score and response to immunotherapy? How can genetic screening be performed on the basis of the IS phenotype? How does T-cell exhaustion affect the formation of the IS? What are the mechanisms by which primary and metastatic cancers resist the formation of IS differs and warrants exploration?

In summary, the significance of IS within the sphere of health and various diseases is attracting increasing interest, and there is a prevailing conviction that scholarly inquiry into this domain will culminate in unforeseen and momentous discoveries in the future.
